# Curation of myeloma observational study MALIMAR using XNAT: solving the challenges posed by real-world data

**DOI:** 10.1186/s13244-023-01591-7

**Published:** 2024-02-16

**Authors:** Simon J. Doran, Theo Barfoot, Linda Wedlake, Jessica M. Winfield, James Petts, Ben Glocker, Xingfeng Li, Martin Leach, Martin Kaiser, Tara D. Barwick, Aristeidis Chaidos, Laura Satchwell, Neil Soneji, Khalil Elgendy, Alexander Sheeka, Kathryn Wallitt, Dow-Mu Koh, Christina Messiou, Andrea Rockall

**Affiliations:** 1 Division of Radiotherapy and Imaging, The Institute of Cancer Research, London, UK; 2 National Cancer Imaging Translational Accelerator, London, UK; 3 Department of Radiology, The Royal Marsden NHS Foundation Trust, London, UK; 4 The Royal Marsden Clinical Trials Unit, London, UK; 5 Joint Department of Physics, The Royal Marsden NHS Foundation Trust, London, UK; 6 Department of Computing, Imperial College London, London, UK; 7 Division of Cancer, Department of Surgery and Cancer, Imperial College London, London, UK; 8 Haemato-Oncology Unit, The Royal Marsden NHS Foundation Trust, London, UK; 9 Department of Radiology, Imperial College Healthcare NHS Trust, London, UK; 10 Department of Haematology, Imperial College Healthcare NHS Trust, London, UK; 11 Research and Development Statistics Unit, The Royal Marsden NHS Foundation Trust, London, UK

**Keywords:** Data curation, Data annotation, Magnetic resonance imaging, Myeloma

## Abstract

**Objectives:**

MAchine Learning In MyelomA Response (MALIMAR) is an observational clinical study combining “real-world” and clinical trial data, both retrospective and prospective. Images were acquired on three MRI scanners over a 10-year window at two institutions, leading to a need for extensive curation.

**Methods:**

Curation involved image aggregation, pseudonymisation, allocation between project phases, data cleaning, upload to an XNAT repository visible from multiple sites, annotation, incorporation of machine learning research outputs and quality assurance using programmatic methods.

**Results:**

A total of 796 whole-body MR imaging sessions from 462 subjects were curated. A major change in scan protocol part way through the retrospective window meant that approximately 30% of available imaging sessions had properties that differed significantly from the remainder of the data. Issues were found with a vendor-supplied clinical algorithm for “composing” whole-body images from multiple imaging stations. Historic weaknesses in a digital video disk (DVD) research archive (already addressed by the mid-2010s) were highlighted by incomplete datasets, some of which could not be completely recovered. The final dataset contained 736 imaging sessions for 432 subjects. Software was written to clean and harmonise data. Implications for the subsequent machine learning activity are considered.

**Conclusions:**

MALIMAR exemplifies the vital role that curation plays in machine learning studies that use real-world data. A research repository such as XNAT facilitates day-to-day management, ensures robustness and consistency and enhances the value of the final dataset. The types of process described here will be vital for future large-scale multi-institutional and multi-national imaging projects.

**Critical relevance statement:**

This article showcases innovative data curation methods using a state-of-the-art image repository platform; such tools will be vital for managing the large multi-institutional datasets required to train and validate generalisable ML algorithms and future foundation models in medical imaging.

**Key points:**

• Heterogeneous data in the MALIMAR study required the development of novel curation strategies.

• Correction of multiple problems affecting the real-world data was successful, but implications for machine learning are still being evaluated.

• Modern image repositories have rich application programming interfaces enabling data enrichment and programmatic QA, making them much more than simple “image marts”.

**Graphical Abstract:**

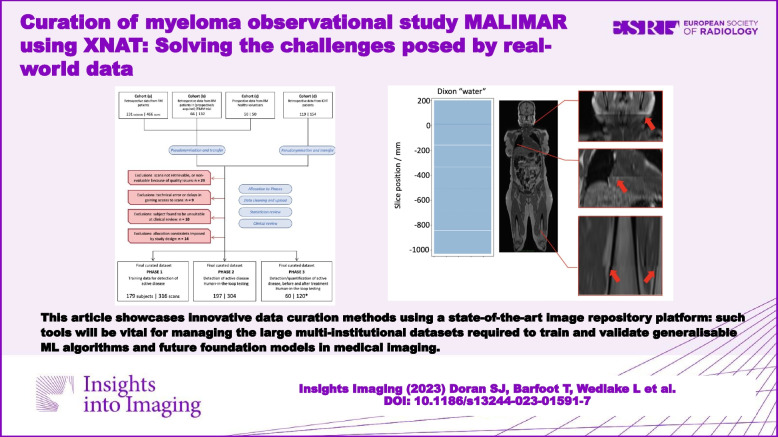

**Supplementary Information:**

The online version contains supplementary material available at 10.1186/s13244-023-01591-7.

## Introduction

In 2016, on the basis of strong literature evidence [1], the UK’s National Institute for Health and Care Excellence (NICE) recommended using whole-body magnetic resonance imaging (WB-MRI) as the first-line imaging tool for diagnosis of myeloma [2]. A consensus from the International Myeloma Working Group agreed that identification of more than two focal lesions larger than 5 mm on MRI should now be used as an indication to treat [3, 4]. An optimised WB-MRI protocol has also been published [5], recommending Dixon and diffusion-weighted MRI (DWI) from skull vertex to knees plus sagittal spine imaging as the basis for disease assessment.

Radiological reporting therefore requires inspection over the whole imaging volume of at least seven different image series: T1-weighted Dixon “in phase”, “out of phase”, “fat” and “water”, two DWI “*b*-values”, and an apparent diffusion coefficient (ADC) map. Patterns of marrow infiltration, burden of disease and degree of response all influence prognosis, but objective quantification is challenging, in principle requiring the generation of large numbers of regions of interest (ROIs) in 3-D on multiple image contrasts and the derivation of quantitative imaging biomarkers.

Manual analysis of this nature is impractical in the clinical workflow, and this is an area that is ripe for the use of artificial intelligence (AI). However, the creation of robust and generalisable automated analysis tools first requires the assembly of large and sufficiently diverse datasets to support model training and validation. Associated clinical data and annotation — consisting of case report forms (CRFs) and images that have been segmented by domain experts — need to be both complete for each subject and also presented homogeneously to AI models. The Machine Learning In MyelomA Response (MALIMAR) study [6], which contains 736 imaging sessions for 432 data subjects, is designed to achieve this ambitious goal. MALIMAR comprises real-world data (RWD) retrospectively sourced at two different hospitals, data from a previous prospective clinical study and images acquired prospectively for MALIMAR on healthy volunteers. It addresses detection, classification, assessment of disease burden and the impact of AI on the radiologist “reading” process.

The importance of data curation is often underplayed in the AI literature [7]. We report here on significant innovations in curation methodologies, made necessary by the diversity of data sources in MALIMAR. The purpose of this article is to demonstrate how automated methods in conjunction with an integrated data repository increased the robustness and quality of data for analysis. Challenges in sourcing and reconstructing historical datasets required development of algorithms to “clean” and “harmonise” data. XNAT helped us to allocate subjects and imaging sessions to phases of the project, to share securely the results of analyses and to perform quality assurance programmatically in a way that was repeatable, auditable and self-documenting.

To date, few machine learning (ML) studies of WB-MRI of myeloma with close to the scope of MALIMAR have been reported in the literature, the nearest comparator being the multicentre pilot analysis of 102 patients recently published by Wennmann et al. [8], which incorporated both automated segmentation and radiomics. Pilot studies [9, 10] recently reported encouraging results for myeloma lesion segmentation in relatively small numbers of cases. Other groups [10–13] have used radiomics methods for myeloma lesion classification on regions of interest (ROIs) already segmented by radiologists. Jerebko et al. [14] developed an early computer-aided detection algorithm for vertebral column metastases in WB-MRI, and, more recently, He and Zhang [15] and Zhou et al. [16] have used deep learning (DL) methods to classify MR images of myeloma patients, but without the use of DW-MRI. A preliminary DL analysis of some of the MALIMAR data, presented by Qaiser et al. [17], assigned disease status to bone regions rather than segmenting individual lesions. Hwang and colleagues [18] considered the problem of fully automated segmentation of bone marrow, accounting for indistinct borders between the bone marrow and other tissues in the presence of disease. We have also previously developed methods for organ localisation in the type of Dixon WB-MRI used here [19, 20], and other modalities have also been investigated [21, 22].

## Materials and methods

### Data sources

Images for the observational MALIMAR study were drawn from two UK NHS institutions, the Royal Marsden NHS Foundation Trust (RM) and Imperial College NHS Trust (ICHT), acquired over the period 2011–2020. Data from four subject populations were included:

- Retrospective clinical data from RM patients
- Data from RM patients acquired as part of the prospective Image-guided Theranostics in Multiple Myeloma (iTIMM) study (ClinicalTrials.gov identifier: NCT02403102)
- Prospective data acquired for the MALIMAR study from RM healthy volunteers
- Retrospective clinical data from ICHT patients

**Fig. 1:**
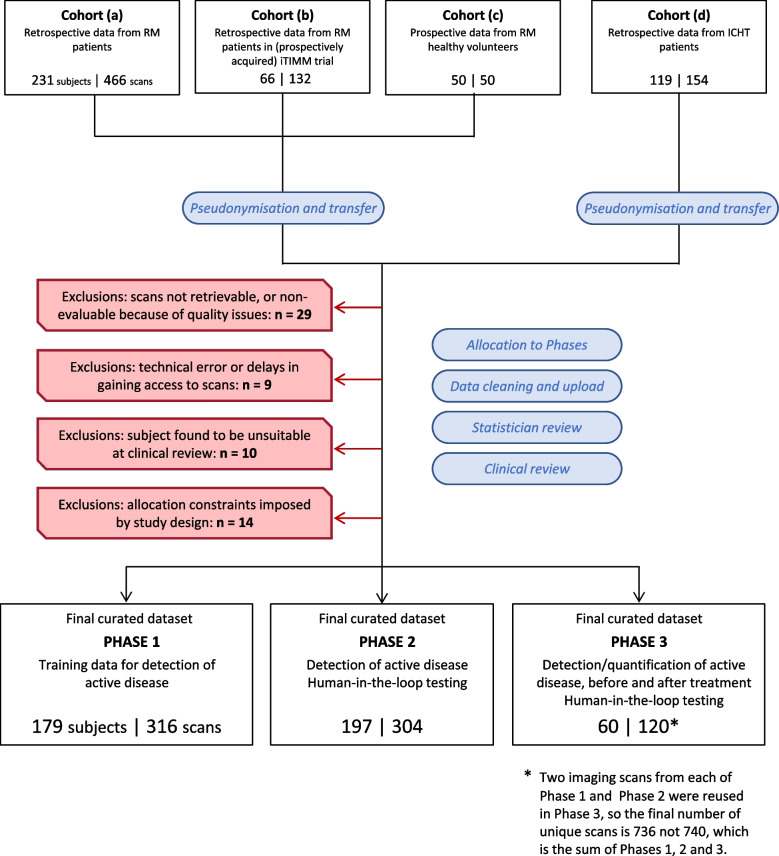
CONSORT diagram for MALIMAR study. Phase 1 scans were for model training, Phase 2 were for human-in-the-loop testing of single time-point WB-MRI scans for detection of active disease and Phase 3 scans were for human-in-the-loop testing of pre- and post-treatment MRI scans for detection of active disease as well as quantification of disease for detection of response

Figure 1 shows the study CONSORT diagram. Note that a number of patients in population (b) also had clinical scans performed outside of the iTIMM trial. When these were used in MALIMAR, this group of patients overlaps with population (a).

### Data acquisition

Data, as originally acquired, consisted of WB-MRI examinations comprising scans at multiple “stations” (patient couch positions). Complete data volumes for further study were created by “composing” (or “stitching together”) data from these stations. All subjects had both Dixon T1-weighted imaging and DWI with multiple *b*-values plus associated ADC map. Supplementary Fig. 1 illustrates the different scanners and protocols, together with the complete curation history and a more fine-grained analysis of exclusions.

### Data transfer and pseudonymisation

Data were pseudonymised at the site of the clinical acquisition and transferred to the XNAT platform at the Institute of Cancer Research. See the supplementary information for details.

### Disease patterns and allocation to project phases

The following presentations of disease seen at imaging were defined: diffuse (D), extramedullary (EM), paramedullary (PM), focal (F) and micronodular (MN). These are not mutually exclusive, and some images show the presence of both focal and diffuse disease. For some patients, disease was considered inactive (I). The study contains both previously treated and treatment-naïve patients, the latter being coded with the “new diagnosis” (N) label. In addition, part of the imaged population is made up of healthy volunteers (H). Given that disease may evolve and that some subjects contribute several sessions to the study, the *pattern* of disease (coded as a combination of the above letters) was assessed at the level of an individual imaging session and could change over time for any given subject. Table 1 shows the distribution of disease patterns, illustrating a large number of potential subpopulations to study, some with low prevalence.

**Table 1:** Disease patterns for Phases 1 and 2, specified on a per-session basis. (Equivalent data are unavailable for Phase 3 at the time of writing). An individual subject may contribute sessions in a number of different categories below

**Disease pattern**	**No. of sessions Phase 1**	**No. of sessions Phase 2**	**No. of sessions Phases 1 + 2**
**F**	96	88	184
**I**	63	95	158
**F D**	49	28	77
**D**	38	32	70
**H**	25	22	47
**F EM**	6	9	15
**D N**	5	8	13
**F D PM**	7	6	13
**F D N**	4	5	9
**F N**	6	2	8
**F PM**	4	2	6
**F PM EM**	2	4	6
**F D PM EM**	5	0	5
**F D EM**	2	1	3
**MN**	2	0	2
**D EM**	1	0	1
**D MN**	1	0	1
**F MN**	0	1	1
**MN N**	0	1	1
**Total**	**316**	**304**	**620**

Key to symbols: *D* Diffuse, *EM* Extramedullary, *PM* Paramedullary, *F* Focal, *H* Healthy, *I* Inactive, *MN* Micronodular, *N* New diagnosis

Analysis was divided into project phases [6], where Phase 1 data are training samples and Phases 2 and 3 contain validation data. For the purposes of allocation between phases, subjects from cohorts (a), (c) and (d) were coalesced into larger categories (focal, diffuse, inactive and healthy — see Table 2). Phase 3 was composed entirely from cohort (b) iTIMM patients, and remaining subjects were allocated as described in the supplementary information.

**Table 2:** Disease categories for Phase 1 and Phase 2, specified on a per-subject basis. (Equivalent data are unavailable for Phase 3 at the time of writing)

**Disease category**	**No. of subjects Phase 1**	**No. of subjects Phase 2**	**No. of subjects Phases 1 + 2**
**D**	73	72	145
**F**	60	62	122
**I**	21	41	62
**H**	25	22	47
**Total**	**179**	**197**	**376**

Key to symbols: *D* Diffuse, *F* Focal, *I* Inactive and *H* Healthy

### Data cleaning

Data cleaning was semi-automatic; a processing script was launched for each imaging session, following which most steps were automatic except where manual intervention (managed by the script) was needed in isolated cases to resolve certain of the correction issues described below. The entire processing chain was recorded as a Jupyter Notebook (see Supplementary Listing 2), and this was subsequently “crystalised” into a noninteractive HTML file and uploaded to XNAT as a “session resource” for the purposes of eventual auditing. The following aspects of the data cleaning are described in detail in the supplementary information:

- Consolidation of data and removal of extraneous MR sequences
- Correction of issues related to the vendor’s “composition” algorithm
- Harmonisation of field-of-view and spatial resolution
- Reformatting coronal Dixon data to transverse to provide a consistent input to ML algorithms
- Data upload to final location

### Phase 1 segmentations

To support the ML objectives of Phase 1, two separate segmentation tasks were performed. A medical physicist (Th.B.) created approximate manual segmentation masks for 18 bony structures on each of a subset of 75 scans, and these served as “bounding regions” to train the algorithm described in [17]. For a sample of 68 sessions allocated to Phase 1, two senior radiologists (A.R., Ta.B.) and two trained radiology registrars (K.E., A.S.) segmented all focal active lesions on the high *b*-value diffusion images. Technical details of the implementation strategy are provided in the supplementary information.

Post-dating the start of MALIMAR, there have been rapid developments in the field of automated segmentation, often based on DL, to the extent that impressive generic tools (e.g. [23, 24]) have now been reported. However, no such tools operating on MR data were available during the design and execution phases of the project.

### Semantic labelling of disease

Each case was assessed by one of four senior radiologists (C.M., D.M.K., T.B. and A.R.), and clinical information was reviewed by one of two senior haematologists (M.K., A.C.). For each of the 18 bony regions, the following annotations were recorded: number of focal lesions, maximum lesion size, the presence of diffuse disease, the presence of inactive disease, whether the region appeared normal and the presence of any imaging artefact. See the supplementary information for further details.

### Data aggregation

XNAT acted as the canonical record for the following data types for individual imaging sessions:

- Disease pattern and radiologist observations
- DICOM source images
- NIfTI representations of the DICOM data for training ML algorithms
- Electronic case report forms (eCRFs)
- Manual segmentations
- Data-cleaning reports
- Administrative data (e.g. completion status of annotations)

At an overall project level, XNAT stored management data and research outputs:

- The project protocol
- Records of case allocations to project phases (both final spreadsheets and the Jupyter Notebooks that created them)
- Python scripts for curating the data
- ML outputs
- Meeting minutes
- Progress reports
- Conference submissions and publications

### Programmatic QA of the entire curation process

Although individual processing steps described were automated, some workflows (e.g. segmentation and semantic annotation) were manual and initiated per session. Initial data gathering was performed by multiple staff members manually retrieving data from PACS, with progress recorded via a set of spreadsheets. Final verification of data integrity and generation of metrics was performed by programmatic analysis of XNAT and spreadsheet data.

## Results

### Image retrieval

It was known at the planning stage of MALIMAR that issues existed with the data composition algorithm. Our initial intention was to retrieve original (uncomposed) DICOM series from the MRI unit’s research DVD archive at RM and to compose these data via an independent algorithm created by the MALIMAR team. However, this exercise was abandoned midway after the discovery that approximately 20% of sessions had incomplete original data (full data availability survey is reported in the supplementary information). The decision was thus taken to work with composed (clinical standard of care) data from PACS, correcting any deficiencies as far as possible.

### Variation in image sequence parameters

**Fig. 2:**
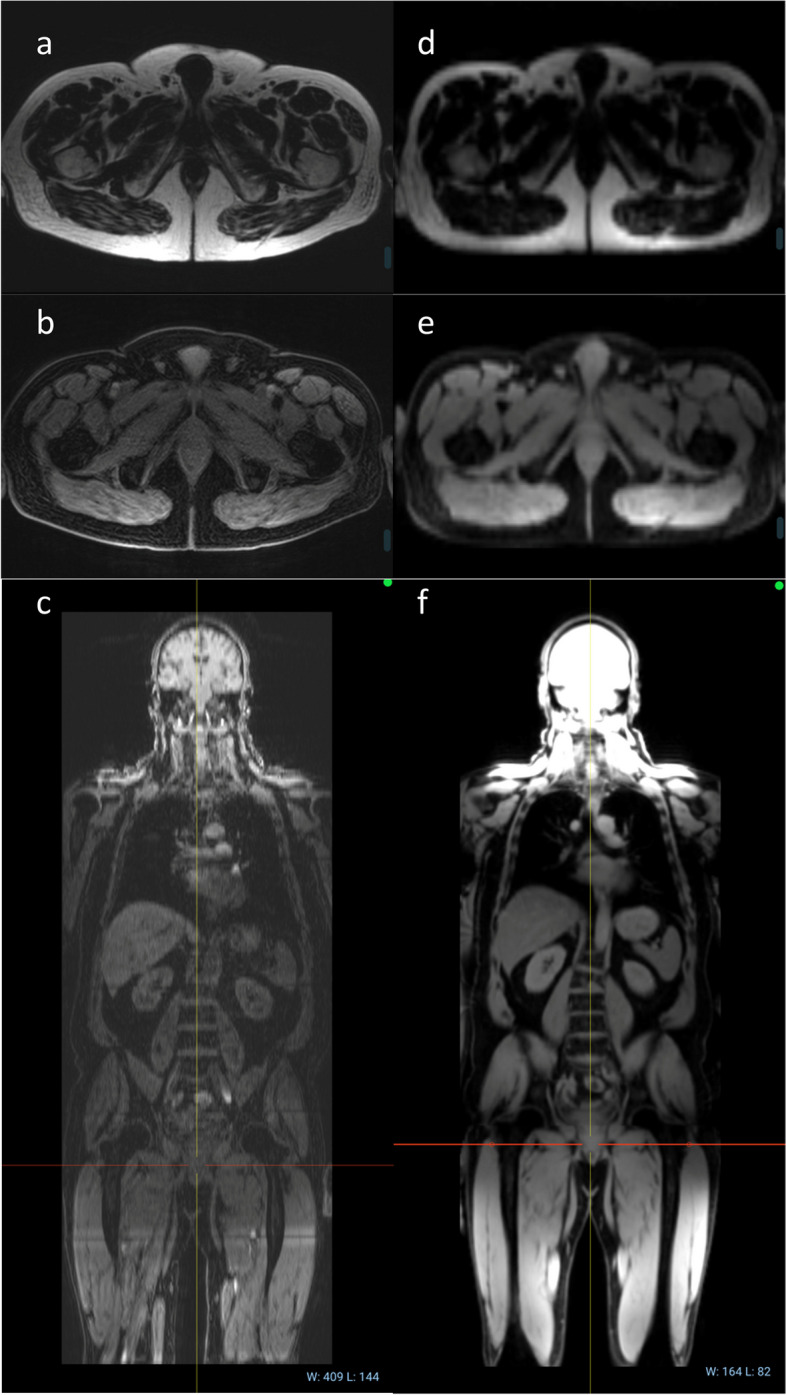
Scans of the same patient made on the RM Siemens Avanto illustrating the two different protocols used in this study: **a**–**c** “transverse” Dixon protocol trades signal-to-noise ratio for increased resolution in transverse slices compared with (**d**–**f**) “coronal” protocol. **a**, **b** and **f** are the acquired series, while **c**, **d** and **e** are reformatted versions of the data

Figure 2 demonstrates the difference between the two different imaging protocols that were in use at the RM for the MAGNETOM Avanto scanner (Siemens Healthcare, Erlangen, Germany). The newer “transverse” Dixon protocol, shown in panels a–c, trades slower acquisition time and poorer signal-to-noise ratio for increased resolution in transverse slices (matching the DWI), compared with the previous coronal protocol (d–f).

### Data cleaning

**Fig. 3:**
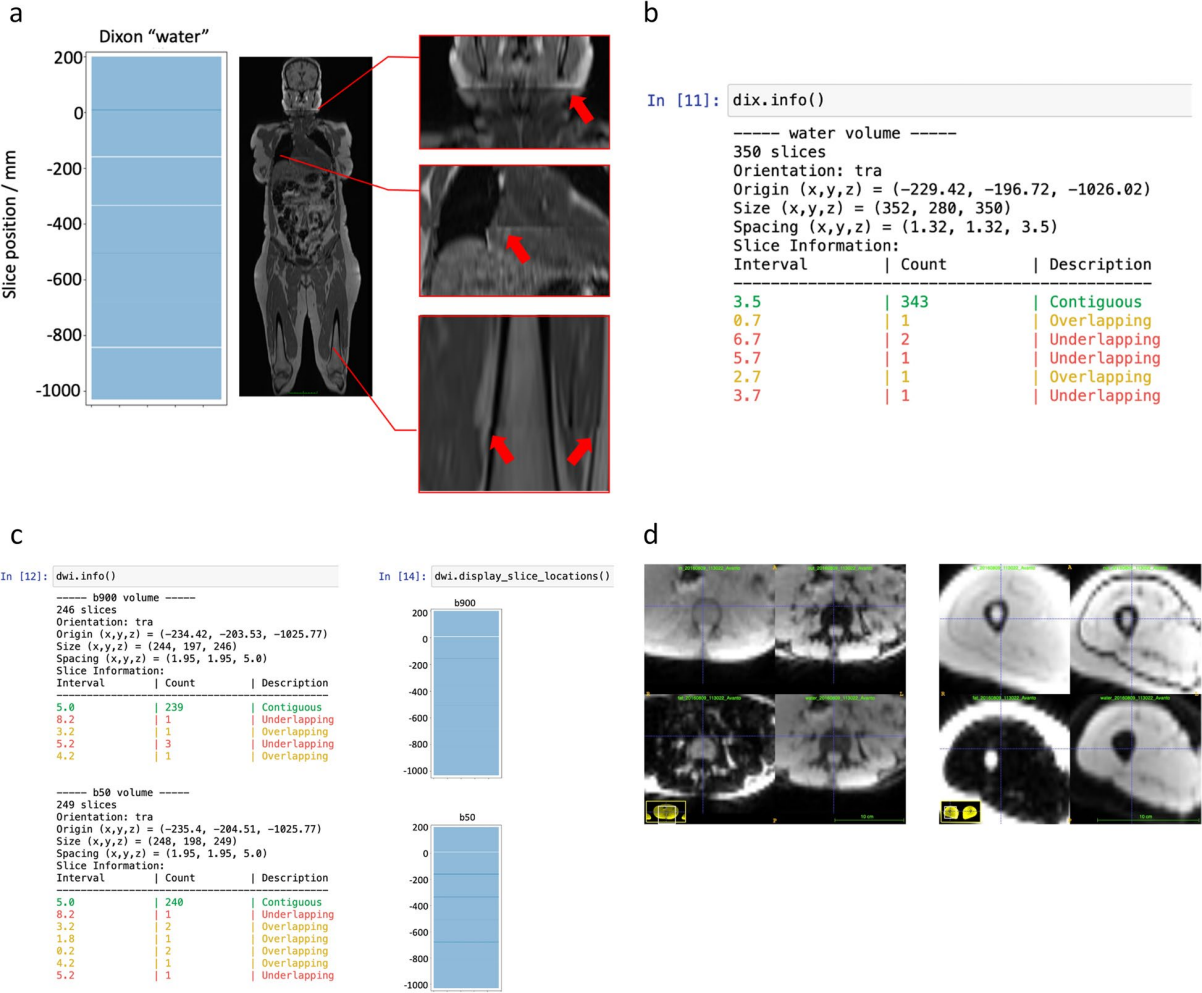
**a** Representation of pattern of slice separation in source data prior to cleaning: light blue, contiguous slice data; white, slice underlap; dark blue, slice overlap. When these axially acquired slices are displayed in a coronal reformatting, image artefacts are evident, and these are highlighted by the arrows in the inset enlargements. **b** Summary report generated by the slice-correction portion of the cleaning algorithm for the “water” images of the same patient as **a**. Note that overlapping slices (amber warning) can be corrected by interpolation, with no loss of information. Underlapping slices (red warning) represent missing data in the “composed” images. **c** Summary report and graphical representation of slice locations for the diffusion data of the same patient as **a** and **b**. Note the difference in underlap and overlap pattern between the composed versions of the two images series acquired with the same fields of view but different *b*-values. **d** Examples from a different patient of the effect of image composition on the positions of corresponding structures on nominally co-registered images. The small inset displays the location of the enlarged image region within the entire slice

Figure 3 illustrates issues encountered with image composition. These were diagnostically insignificant but problematic for ML training data. Figure 3a is a graphical representation of incorrect slice spacings for a Dixon “water” dataset from PACS. Arrows in the inset enlargements demonstrate their impact. 3b is the corresponding summary report generated by our Python cleaning code. 3c illustrates how slice-spacing errors in the b50 and b900 data from the same patient could differ both from the Dixon data and from each other. In 3d (for a different patient), both the “water” and “fat” images have been reconstructed from the *same* in- and out-of-phase Dixon data and should be inherently co-registered. However, it is evident that the composition algorithm introduces an offset such that corresponding structures are not aligned. Moreover, this within-slice shift is not consistent even within a single patient but varies between stations.

Supplementary Figs. 2, 3, 4 and 5 provide further examples of data quality issues. The so-called fat-water swaps were encountered in approximately 10% of the imaging sessions used. These typically took one of three forms:

- Global mislabelling — Composed series labelled “fat” actually contained the Dixon “water” images and vice versa.
- Station mislabelling — DICOM series for one or more of the original stations were labelled “fat” when actually containing the Dixon “water” images and vice versa, thus leading to a composed image in which one or more blocks had the wrong contrast.
- Local fat-water swap — A region within the volume of a single station was misidentified. Supplementary Fig. 5 illustrates this and also demonstrates the impact of implanted metal on Dixon and diffusion-weighted imaging.

For MALIMAR, the most expedient route to solve this issue was exhaustive checking of data by a member of the research team. However, recent work [25] suggests the possibility of automated detection and correction of the problem.

Several other types of artefacts were also observed, including motion, distortions and abnormally low image signal-to-noise ratio.

### Results of segmentation

**Fig. 4:**
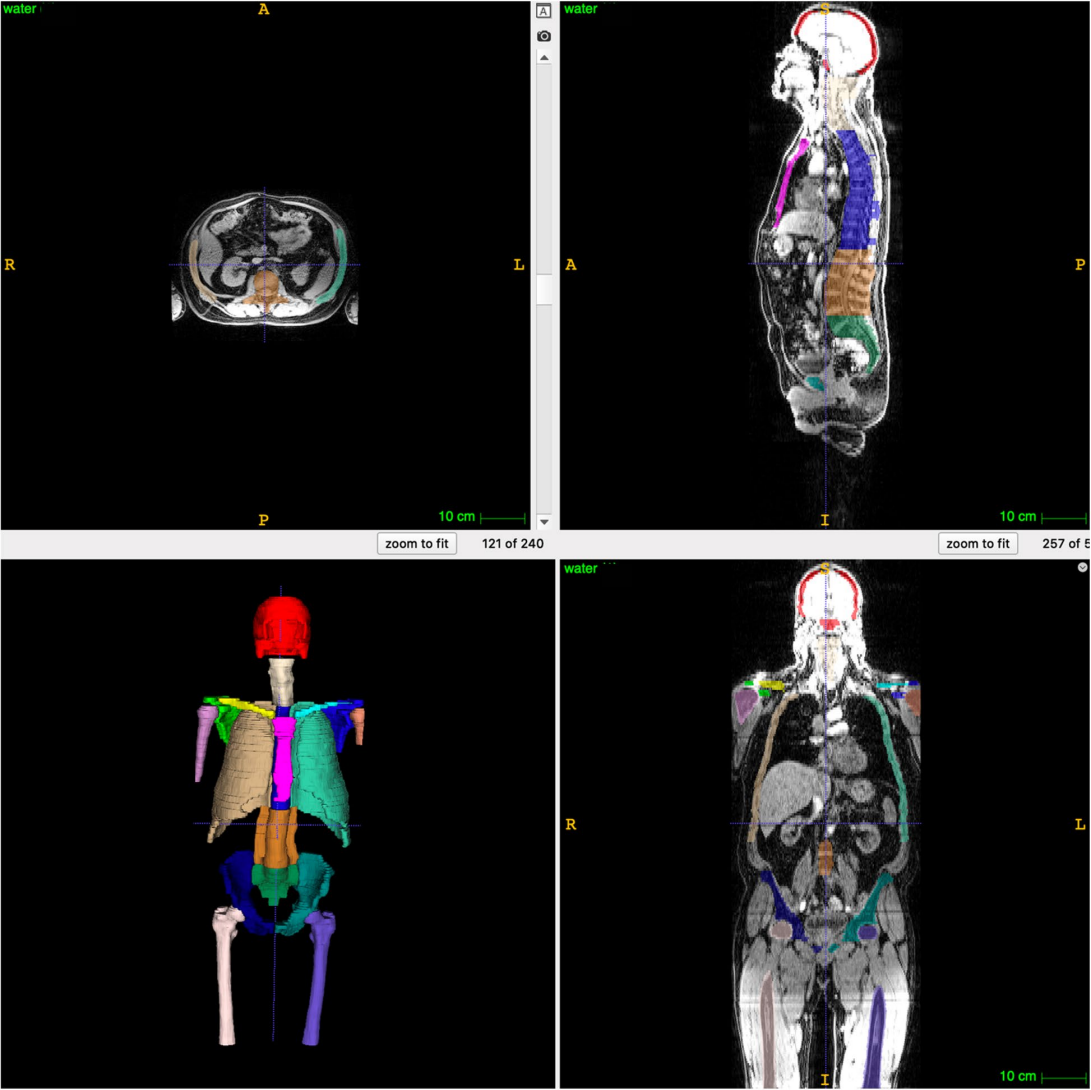
Example of an image segmentation (performed on the dataset of Fig. 2b and c) for MALIMAR Phase 1 as training data for an auto-segmentation routine. Note the compromise solution of a crude spine and rib segmentation, rather than a detailed segmentation of individual vertebrae

**Fig. 5:**
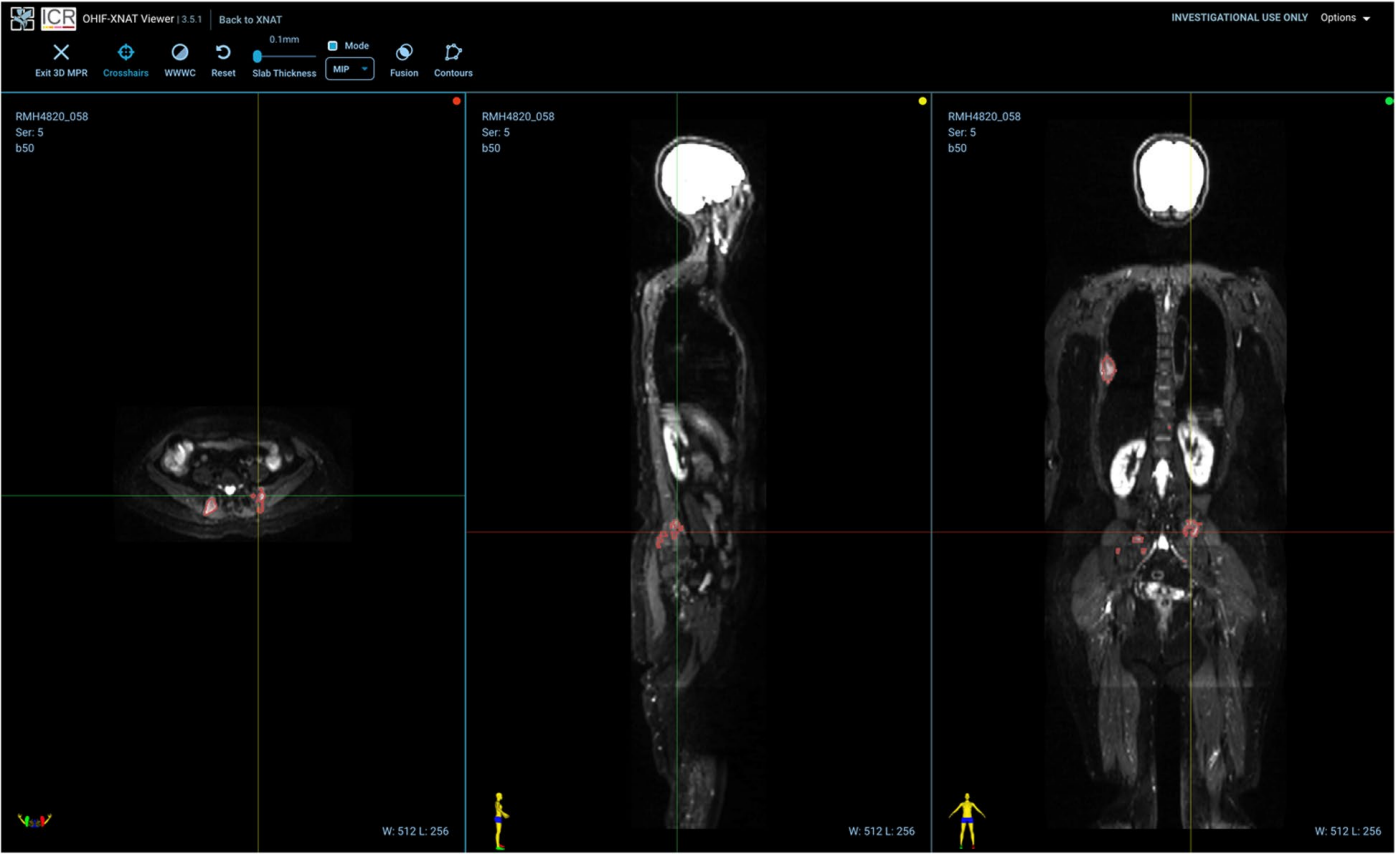
Example of an image segmentation of focal lesions (for a different patient from Figs. 2, 3, 4)

Figure 4 shows a visualisation of the results of manual segmentation of the bony structures. The results of the automated procedure are presented in [17]. Figure 5 illustrates focal lesion segmentation.

## Discussion

MALIMAR combines prospectively acquired data, with a locked-down acquisition protocol, and variable “real-world” data, whose use presents consistency challenges for ML [26]. Incompatibility between MRI data sources arises because of the following:

- It may be impossible to match protocols on a scanner from one vendor with exact equivalents from another.
- Even from the same vendor, hardware and software capabilities vary between models (and sometimes within the same model).
- Scanners and software versions are updated.
- Knowledge, experience and clinical requirements evolve, leading to the use of different sequences.
- Logistical considerations dictate particular acquisition strategies in the clinic, and these may be different between study sites.
- Protocols may mandate patient coverage (e.g. from skull vertex to knees in myeloma) rather than field of view, and so the number of stations may vary between patients of different heights.
- Although not relevant for MALIMAR, running an identical pulse sequence at different field strengths may give rise to different image contrasts.

Thus, it is unrealistic for machine-learning researchers to expect real-world, multi-institutional data to be *a priori* compatible, and this leads to the need for significant curation and pre-processing.

Variable provenance means that anonymisation strategy and methods of data upload need careful thought, and this is highly relevant for large multi-national and multi-institutional data curation efforts currently underway (e.g. The Cancer Imaging Archive (TCIA) and Imaging Data Commons (IDC) in the USA and European Union Horizon 2020 projects CHAIMELEON, EuCanImage, ProCAncer-I and the recently inaugurated European Federation for Cancer Images (EUCAIM) https://www.egi.eu/project/eucaim/).

Curation strategies also need to be flexible enough to respond to situations that are discovered only after the study design has been finalised. MALIMAR exemplifies how, in the “AI era,” routine patients become research subjects retrospectively. Data are used for purposes that not only differ from the primary healthcare need but also had not even been formulated at the time of data acquisition. Such projects must be conducted within an appropriate ethical framework, and guidance has been issued by the UK’s Royal College of Radiologists [27]. There are increasing incentives for institutions to duplicate all future patient data to a research archive separate from the hospital PACS. Indeed, many institutions — and even entire nations [28] — are going further and creating research copies of their entire historical PACS.

Correcting data to compensate for errors in the “composition” step consumed a significant length of time, and our findings argue strongly for retaining all original data. However, this research requirement has consequences for the clinical reporting workflow: either the PACS becomes cluttered or hanging protocols need to display only the relevant series in an appropriate layout to meet the needs of the reporting radiologists.

It was *a priori* undesirable that patients were scanned with two substantially different imaging protocols (transverse and coronal Dixon images, as illustrated in Fig. 2). However, given that the coronal protocol accounted for almost 30% of sessions, it was deemed not feasible to remove these scans. The supplementary information lists strategies we considered to overcome this problem.

Curation via programmatic means aids repeatability and can be made self-documenting. Automating the allocation of imaging sessions between project phases allowed us to fine-tune a complex algorithm and implement it without the risk of errors arising from difficult-to-replicate manual interventions. Via our novel combination of script and data platform, we aimed to make it straightforward to reanalyse the entire study from scratch, in the light of new methods or knowledge, with minimal human interaction, in a time governed only by computational power and data throughput. Given this ability, MALIMAR provides a highly useful dataset and framework for future work to isolate the impact of each of the data curation procedures on downstream machine learning models.

MALIMAR revealed significant shortcomings in data archiving that had existed historically even in a research-active institution with an excellent MRI department. The data spanned an era in which the problem of a large (many thousands of DVDs) research data archive was already being addressed, and the findings reflect limitations of the technology available at the time, which were incompatible with the pressures of a busy clinical unit. These historical archiving practices and the associated problems are not unique to RM and will be relevant for other projects using retrospective data, especially WB-MRI.

A limitation of the study is that all scanners were from the same manufacturer and had the same field strength.

## Conclusions

The MALIMAR project addresses a problem of significant unmet clinical need for which there was no large pre-existing curated dataset. We have demonstrated how multi-institutional retrospective data, acquired on different scanner models and over an extended time window, give rise to “real-world” problems. Attention to detail is needed to maximise the utility of the data, but this task can be made easier by automated processing and programmatic QA. MALIMAR showcases the benefits of using a repository platform such as XNAT as an aggregator of data that eases the day-to-day management of multicentre trials, facilitates data sharing with robust access controls and enhances the quality and value of data. All of these aspects will prove vital in the coming years as the size and complexity of image datasets increase and as new data repositories at national and transnational scales come on-stream.

### Supplementary Information

**Additional file 1: Supplementary Video 1.** Complete 3-D data for reformatted and “composed” image series, illustrating a case where there are severe slice contiguity issues. Uncorrected, as here, the images would act as confounders for training ML algorithms.

**Additional file 2: Supplementary Video 2.** Partially corrected 3-D data for the case shown in Supplementary Video 1. Where data are missing then the correction is imperfect.

**Additional file 3:** **Supplementary Information.** Additional details clarifying and extending the descriptions in the main text. **Supplementary Figure 1.** Detailed CONSORT diagram for MALIMAR study, augmented with data processing details and intermediate staging points. Phase 1 scans were for model training; Phase 2 were for human-in-the-loop testing of single time-point MRI scans for detection of active disease and Phase 3 scans were for human-in-the-loop testing of pre- and post-treatment MRI scan for detection of active disease as well as quantification of disease for detection of response. **Supplementary Figure 2.** “Poster frame” accompaniment to videos of complete 3-D data for reformatted “composed” image series, illustrating a case where there are severe slice contiguity issues. Uncorrected, the images would act as confounders for training ML algorithms. The original images can be partially corrected, but if data are missing then the correction is imperfect. **Supplementary Figure 3.** Reformatted “composed” image series from a patient illustrating a case with completely misordered station data. **Supplementary Figure 4.** Reformatted “composed” image series for two patients showing examples of the variations in field-of-view encountered. **Supplementary Figure 5.** Example of image with severe, but isolated, artefact due to presence of metal, with (inset) the corresponding b50 diffusion-weighted image. Other parts of the 3-D dataset may be suitable for machine learning. Note also (arrows) a local fat-water swap in the Dixon reconstruction. **Supplementary Listing 1.** Jupyter notebook illustrating the algorithm used for allocating imaging sessions to different trial phases and the way that this was made “self-documenting”. Due to constraints of trial management, the RMH and ICHT data were assigned as separate processes. **Supplementary Listing 2.** Jupyter notebook illustrating the algorithm used for cleaning imaging sessions and the way that this was made “self-documenting”. Note that the script refers to a set of underlying Python objects that were developed using a traditional Python coding methodology with the PyCharm IDE. At the point marked *, the Jupyter script launches an interactive editing tool to correct for image shifts. Arrows represent the order of the process flow.

## Data Availability

Reasonable requests for access to the data described in this article will be considered by the Trial Steering Committee and should in the first instance be addressed to the first author. For further information on any of the methods reported here, or access to any of the data curation source code, please contact the first author.

## Abbreviations

ADC: Apparent diffusion coefficient
AI: Artificial intelligence
CONSORT: Consolidated Standards of Reporting Trials
CRF: Case report form
DICOM: Digital Imaging and Communications in Medicine
DL: Deep learning
DVD: Digital video disk
DWI: Diffusion-weighted imaging
eCRF: Electronic case report form
EUCAIM: European Federation for Cancer Images
ICHT: Imperial College NHS Trust
IDC: Imaging Data Commons
iTIMM: Image-guided Theranostics in Multiple Myeloma (a clinical study)
MALIMAR: Machine Learning In MyelomA Response (a clinical study)
ML: Machine learning
NICE: The UK’s National Institute for Health and Care Excellence
NIfTI: Neuroimaging informatics technology initiative
PACS: Picture archiving and communications system
RM: Royal Marsden Hospital
ROI: Region of interest
TCIA: The Cancer Imaging Archive
WB-MRI: Whole-body magnetic resonance imaging
