# Supplementary material for: Curation of myeloma observational study MALIMAR using XNAT: solving the challenges posed by real-world data
Source: Insights Imaging. 2024 Feb 16;15:47. doi: 10.1186/s13244-023-01591-7 (PMC10869673; doi:10.1186/s13244-023-01591-7)
Supplement: Supplementary file 3 — Additional file 3: Supplementary Information. Additional details clarifying and extending the descriptions in the main text. Supplementary Figure 1. Detailed CONSORT diagram for MALIMAR study, augmented with data processing details and intermediate staging points. Phase 1 scans were for model training; Phase 2 were for human-in-the-loop testing of single time-point MRI scans for detection of active disease and Phase 3 scans were for human-in-the-loop testing of pre- and post-treatment MRI scan for detection of active disease as well as quantification of disease for detection of response. Supplementary Figure 2. “Poster frame” accompaniment to videos of complete 3-D data for reformatted “composed” image series, illustrating a case where there are severe slice contiguity issues. Uncorrected, the images would act as confounders for training ML algorithms. The original images can be partially corrected, but if data are missing then the correction is imperfect. Supplementary Figure 3. Reformatted “composed” image series from a patient illustrating a case with completely misordered station data. Supplementary Figure 4. Reformatted “composed” image series for two patients showing examples of the variations in field-of-view encountered. Supplementary Figure 5. Example of image with severe, but isolated, artefact due to presence of metal, with (inset) the corresponding b50 diffusion-weighted image. Other parts of the 3-D dataset may be suitable for machine learning. Note also (arrows) a local fat-water swap in the Dixon reconstruction. Supplementary Listing 1. Jupyter notebook illustrating the algorithm used for allocating imaging sessions to different trial phases and the way that this was made “self-documenting”. Due to constraints of trial management, the RMH and ICHT data were assigned as separate processes. Supplementary Listing 2. Jupyter notebook illustrating the algorithm used for cleaning imaging sessions and the way that this was made “self-documenting”. Note that [file 13244_2023_1591_MOESM3_ESM.pdf]

Curation of myeloma observational study MALIMAR using XNAT:
Solving the challenges posed by real-world data

ELECTRONIC SUPPLEMENTARY MATERIAL

1. Data acquisition

Acquisition at the Royal Marsden NHS Foundation Trust (RM) took place on two different scanners, a MAGNETOM Aera 1.5 T and a MAGNETOM Avanto 1.5 T (Siemens Healthcare, Erlangen, Germany), whilst at Imperial College NHS Trust (ICHT), a MAGNETOM Aera 1.5 T was used. Although some imaging protocols pre-date the publication of the MY-RADS guidelines [1], all followed the overall acquisition scheme described in MY-RADS. However, given that many of the patient data used were collected as part of standard-of-care imaging, the exact sequences and parameters used varied according to the local protocols in place at the two institutions at the time of data acquisition, leading to differences in slice thickness, in-plane resolution, flip angle and repetition time.

2. Creation of composed images

Magnetic Resonance Imaging (MRI) scanners have a field-of-view that is limited in the head-foot direction. Thus, in order to obtain whole-body images, it is necessary to acquire a number of separate imaging volumes — between three and eight for the protocols used in MALIMAR — each with the patient bed in a different location ("station"). The DICOM series corresponding to these stations are then "composed" (or "stitched together"), either manually by radiographers at the scanner console or by a proprietary manufacturer algorithm running on the scanner that also performs various image-processing steps, such that in both cases a single resultant DICOM series contains a complete whole-body image.

In common with many other centres at the time of acquisition, local practice at the RM was to send only localisers, sagittal spine images and composed whole-body DICOM series to the Picture Archiving and Communications System (PACS) for radiological review. In principle, the complete datasets as acquired (including all the original stations for Dixon and DWI) were transferred to a digital video disk (DVD) archive maintained for research purposes.

Data from ICHT were sourced from the institution's PACS, where some were available with all the original imaging stations separately and some only as composed images.

3. Pseudonymisation, data transfer and data versions

For the initial data-aggregation step at RM, images from either PACS or DVD were transferred via the DICOM protocol to an XNAT instance running within the RM clinical firewall, which contains patient-identifiable data, leading to RM/ICR Version 1 of the study dataset. A shell script, running on this server, incorporated a call to DicomRemap (Computational Imaging Lab, Washington University, St Louis, MO, USA) to batch-pseudonymise data for multiple patients. DicomRemap invokes an anonymisation script written in the DicomEdit language [3] and, for MALIMAR, we wrote a script that implemented the parts of the DICOM Standard Part 3.15 Annex E Basic Application Level Confidentiality Profile with Retain UIDs option that were relevant for our MRI data types. Data were then sent to an XNAT instance, (accessible only from the Institute of Cancer Research (ICR) internal network) for further processing (RM/ICR Version 2).

ICHT data were retrieved from PACS onto an internal workstation (ICHT Version 1) and pseudonymised using DicomBrowser (Computational Imaging Lab, Washington University, St Louis, MO, USA [4]), using an anonymisation script written for a previous multicentre project [2]. Scans were anonymised individually and manually checked to ensure no patient identifiers were present in private tags. Data were then aggregated onto an encrypted hard disk (ICHT Version 2) for transfer between institutions.

After data curation had been performed, as detailed in the article main text and below in this document, images and clinical data were moved to their final location on a third XNAT instance, also installed within the ICR but visible outside the ICR network, from which suitably authorised research "end users" could download data for onward processing, e.g., for machine learning. Version 3 of the data combined the full multi-institutional data and also incorporated all the manual segmentations, eCRFs and machine learning outputs, thus demonstrating the "added value" created by the project.

See Supplementary Figure 1 for a graphical representation of this process.

4. Allocation of cases between project phases

For reasons connected with management of the study, the allocations between project phases for the data from RM and ICHT were performed at separate times. Phase 1 acts as a training dataset, whilst Phases 2 and 3 are validations. The allocation algorithm was as follows:

1. Allocate all Cohort (b) iTIMM patients to Phase 3.
2. Condense the disease patterns described in the main text into a simpler four-way classification (disease on a *per-patient* basis of focal, diffuse, inactive or healthy (F,

D, I, H) on the basis of the following rules, as applied to the first scan for each subject in the study:

- Any patient with the Inactive label remains as Inactive.
- Any patient with a pattern containing Diffuse is assigned as Diffuse.
- Micronodular disease is assigned as Diffuse.
- All remaining patterns (e.g., F and F EM) are assigned as Focal.

3. For each classification from Step 2, allocate patients from Cohorts (a), (c) and (d) alternately to Phase 1 and Phase 2 by the date of the patient's first whole-body MRI scan in the MALIMAR study. This means that healthy volunteers were split equally between Phases 1 and 2.
4. Allocate only one scan per disease pattern per phase for a given patient to avoid bias if a particular patient has a large number of scans within a short time period.

Note that there was the potential for overlap between patients in Cohorts (a) and (b), as some had standard-of-care imaging unrelated to the iTIMM trial. In four cases, the programmatic check designed to prevent patients being duplicated between phases was inadvertently not run. Images for two patients were thus accidentally present in both Phases 1 and 3 at the time of data analysis, whilst a different pair was duplicated between Phases 2 and 3. Nevertheless, no imaging session was used both for algorithm training and testing, and we believe that no bias occurred because of this oversight.

Information was captured on the relative number of "coronal" vs "transverse" Avanto imaging sessions in Phases 1 and 2, together with histograms of patient age but the allocation algorithm did not use these variables.

Finally, the allocations were written to spreadsheets that provide the inputs to the data cleaning script.

Supplementary Listing 1 provides further details of the algorithm and its implementation using a self-documenting Jupyter notebook.

5. Data cleaning

The Python code makes extensive use of the XNATpy Python application programming interface (API) library (https://xnat.readthedocs.io/) [3], which in turn submits queries to XNAT's representational state transfer (REST) API [4,5]. The data cleaning process is illustrated by the flow chart of Supplementary Figure 1 and consists of the following steps.

Consolidation of data and removal of extraneous MR sequences

A consequence of the different sources of image data was that the MR sequence names and DICOM series instance number varied widely. The content of imaging sessions also varied in terms of both the number of stations and the presence of additional sequences not required by the MALIMAR protocol. The cleaning software read in the data either from the second XNAT instance, visible within ICR only, or from encrypted disk (ICHT data). Data were checked for missing or duplicated series, and, where images were not already supplied as “composed” (“stitched”) series, our software consolidated all stations into a single DICOM study for each image contrast type.

Correction of issues related to manufacturer “composing” algorithm

One of the most complex problems we faced in the project was the accurate reconstruction of the multiple imaging stations into a single 3-D image for each contrast. In principle, this operation had already been performed at source. However, we observed that the vendor-supplied processing used clinically performed sub-optimally for our research. The following issues were noted, with variable incidence between scanners, subjects and imaging sessions:

- incorrect slice ordering.
- non-contiguous data caused by missing slices and slices separated by more than the primary sampling interval (slice underlap); slices separated by less than the primary sampling interval (slice overlap); and duplicated slices.
- within-plane slice shifts such that the effective origin varied between stations.
- shifts between different series that should match (e.g., between the fat, water, in- and out-of-phase Dixon scans, or between different b-values for the same diffusion imaging experiment).

Where possible, specific mitigations were coded for these problems as part of the cleaning algorithm.

Supplementary Figure 2 (corresponding to supplementary Video 1 and Supplementary Video 2) and gives a further examlple to slice contiguity-related issues in the input data, and Supplementary Figure 3 shows how the data composition algorithm can sometimes switch entire stations. Supplementary Figure 4 shows how the data composition algorithm sometimes produced outputs with incorrect fields-of-view.

Harmonising field-of-view and spatial resolution

This took place in two distinct steps. Firstly, at the start of the slice contiguity correction, fields-of-view were harmonised between all the Dixon scans and, separately, all the DWI scans,

such that only the volume common to all the data within each group was retained. Secondly, after registration errors had been corrected for Dixon and DWI groups separately, the Dixon and DWI fields of view were themselves harmonised, with appropriate resampling of the original data where necessary.

Reformatting coronal Dixon data from Avanto

As is evident from Figure 1, a significant fraction of the source data (132/449 patients (30%)) came from a period when patients were scanned at RM using a protocol where Dixon scans were acquired as a coronal dataset. The appearance of the resultant images, consequences for the later manual segmentation step and implications for the downstream machine learning are discussed below, but at this stage, our code simply resampled the source data such that each DICOM slice had axial instead of coronal orientation, with low in-plane resolution in the anterior-posterior direction, but a larger number of slices and a much lower slice thickness than that of the natively acquired axial Dixon scans from the other protocols.

Upload of data

The uniform pre-processing of the data ensured that, in what was uploaded to the destination XNAT instance, every imaging session was represented by a uniform set of four Dixon series (labelled simply as “in”, “out”, “fat” and “water”) and either three or four DWI series (“b50”, [“b600”], “b900”, “adc”) depending on the data source.

6. Phase 1 segmentations

In order to answer the primary research question of Phase 1 of the project, it was necessary to provide, as input data, segmentation masks for the following 18 bony structures: skull, scapula (right and left), clavicle (right and left), manubrium and sternum, spine (upper, middle, lower), ribs (right and left), iliac blade (right and left), sacrum, femur (right and left) and humerus (right and left). The relative merits were discussed, with relation to the eventual task of the machine-learning disease detector, of performing detailed but lengthy segmentations, highly conformed to the structures, vs cruder but much quicker to generate “bounding boxes”. A compromise solution was adopted in which 75 scans were manually segmented by a medical physicist (ThB) to a moderate degree of accuracy, with spinal and rib elements segmented crudely as single units rather than outlining each vertebra and rib. These formed a sufficient “bounding region” training set for a specially-developed ML model to segment the remaining Phase 1 cases automatically. That work, using the DeepMedic software framework, is described in [6] and produced output in the NIfTI file format [7,8], which has become a *de facto* standard for much of the machine-learning community in medical imaging. Finally, we “closed the loop” by converting the NIfTI results back into DICOM segmentation objects such

that they could be viewed on the XNAT platform, overlaid onto the original, untransformed DICOM images.

Similar methods were used for a rough manual segmentation of the focal lesions.

The MALIMAR project ran in parallel with the development of the ICR-OHIF-XNAT viewer [9]. By the end of the curation project described here facilities had become available within the XNAT platform itself both to draw and save 3-D masks and to create electronic case report forms (CRFs). However, these were not available at the outset and so external tools were integrated with XNAT as follows. A Python controller script managed the download of images from XNAT to a temporary directory and used this as the input to an automatically-launched ITK-SNAP [10] session, which was appropriately configured by the Python controller to present the data in a suitable format for labelling. When the user was happy with the label maps created, these were saved and the Python controller automatically uploaded the saved labels back to the correct MR session in XNAT. A radiologist checked all manual segmentations of the skeleton using this same system.

For segmentation of focal lesions, all lesions segmented by registrars (KE and AS) were checked and edited if needed by senior radiologists (AR and TaB). Segmentations were guided by the ground truth case report forms provided by expert reader and clinician (see below for the establishment of ground truth).

7. Semantic labelling of disease

For each of the 18 bony structures described in the previous section, the following annotations were recorded on the case report form (CRF): number of focal lesions as a categorical entry (1–4, 5–10 or >10), maximum lesion size as a categorical entry (0, <10 mm, 10–20 mm, >20 mm), presence of diffuse disease, presence of inactive disease, whether the region appeared normal, and presence of any imaging artefact. A free-text field on the form also allowed radiologists to add additional notes. Some of these proved very useful in the project reconciliation phase, allowing the team to understand some of the more complex assignments of disease pattern.

Radiologists undertaking disease labelling used their standard PACS reporting environment to view the images, and two methods of annotating the data were made available:

i. radiologists filled in a paper CRF template manually and this was subsequently transcribed — in the first instance manually, but later via an automatic image processing interpretation — to an Excel spreadsheet and uploaded to XNAT at the imaging session level.

ii. an automated system, driven by a Python script, that retrieved a template eCRF for disease labelling from XNAT, launched Excel, detected completion of the document by the radiologist and uploaded the spreadsheet to XNAT.

For recording the sites and size of disease on the scans, for the purposes of ground truth, different readers preferred different styles of CRF. Some radiologists found paper forms quicker, whilst others were very happy using the Python + Excel automated workflow that automatically uploaded the data to XNAT.

The scan dates, patient sex and age were originally transcribed manually into spreadsheets from hospital systems. As one of the quality control checks, these spreadsheet dates were compared against the scan dates in the DICOM file metadata.

The only clinical data included in the MALIMAR retrospective study are the scan dates, the patient sex and age at enrolment. Information about the disease presence in different bony structures, and overall assessment of disease category and disease pattern, was generated specifically for the study on the already-curated images (i.e., after exclusions) and so not affected by the use of real-world data. For Phase 3, the categorisation of response from baseline to post-treatment scan was provided by the reference standard from the source study.

The disease pattern and category, the Dixon image orientation and other administrative information relating to the processing status of each imaging session were also stored as XNAT custom variables that were set manually. This led to occasional (< 10) issues where the results of second opinions in difficult cases were not correctly reflected between the archived eCRF spreadsheet and what was displayed in the XNAT UI. Issues were resolved at the end of the study via the Python resolution tool and other checks by the study statistician.

As of release 1.8.8, XNAT now supports the creation of custom forms. Entries in custom forms can be accessed programmatically, thus presenting additional possibilities for automating workflows. Were we to run the same study again, the eCRF workflow would be much simplified by using a custom form to capture all the required data, obviating the need for the spreadsheet.

8. Potential strategies for mitigating the impact of sequence variability

The impact of sequence variability and contrast differences across stations was discussed at a number of MALIMAR steering group meetings. The position of the MALIMAR data curation team was that image intensity harmonisation constituted a “downstream” signal-processing activity that was the remit of the team performing the machine learning, whereas the original

contrast characteristics of the MR data should be retained in our “canonical” version distributed to ML researchers. On the other hand, the removal of obvious errors, such as those introduced post-acquisition by the vendor composing algorithm, was seen as part of the remit of the data curation.

We considered four approaches to how to handle the two substantially different imaging protocols (transverse and coronal Dixon images, as illustrated in Figure 2):

(a) Use separate ML models for the transverse and coronal data.

(b) Use some form of style transfer deep-learning network [11] (e.g., a generative adversarial network (GAN) [12,13]) to transform the coronal images to “pseudo-transverse” ones — although [14] suggests that caution is needed.

(c) Do nothing and hypothesise that the transverse reformatting of coronal data may, in fact, augment the originally transverse images and make the eventual ML algorithms more robust to a variety of inputs.

(d) Adopt a “traditional image processing approach”, based on interpolation to a common spatial resolution, followed by histogram matching [15]. This was the method used by the study team in [6].

Notice, too, that even for the transverse datasets, in-plane resolution varied between the two institutions. It is unclear how these issues will affect machine learning outputs. In the adjacent field of radiomics, for example, significant efforts are underway to examine the differences in texture introduced when images are resampled to a common resolution [16,17], as was performed in our subsequent ML pipeline [6].

9. Programmatic curation of final dataset

In order to reconcile the multiple different data sources of data, a reconciliation application was programmed in Python to accomplish the following functions:

- Combine data from all spreadsheets with live management data retrieved from XNAT (via its REST API) into a single Pandas data frame using the image session ID as primary key.
- Resolve inconsistencies between input data.
- Define a set of “issues”, each tied to a single row in the data frame, providing a categorisation of (a) the category of problem encountered, (b) full explanatory text, and (c) the resolution (e.g., exclusion of the session).

- Codify the division of patients and volunteers between cohorts, categories and study phases.
- Automatically calculate all the totals and exclusions provided in Figure 1, Table 1 and Supplementary Figure 1.
- Define a set of “unit tests” such that whenever an issue was updated, checks were performed to ensure the reported statistics remained self-consistent.

10. Analysis of retrieval success from RM DVD archive

For the iTIMM study:

- 4 out of 124 patient datasets (3%) could not be retrieved from DVD.
- 1 DVD was missing from the archive.
- 5 DVDs had incomplete data. This occurs when a DVD runs out of space during the archiving process and should be flagged to the radiographer by the system, at which point the correct procedure is for the session to be re-archived on a fresh DVD and records updated.
- 4 manually annotated DVDs had incorrect numbers due to confusion between digits (e.g., 1 and 7).

Attempts to retrieve the “research” sequences for the RM routine clinical patient data resulted in the following:

- 36 of 139 datasets (26%) were incomplete with respect to the non-PACS “research” data that included the separated image stations.
- 20 scans (14%) were not on the expected disks.
- 1 DVD could not be read due to damage.

After a lengthy process of detective work, several of the missing datasets were recovered from other disks but 22 DICOM sessions (16%) remained incomplete.

Curation of the ICHT data also found incomplete datasets but a comprehensive survey was not performed.

11. References

1. Messiou, C., et al., *Guidelines for acquisition, interpretation, and reporting of whole-body MRI in myeloma: myeloma response assessment and diagnosis system (MY-RADS).* Radiology, 2019. 291(1): p. 5-13.

2. Rockall, A.G., et al., *Development and Evaluation of Machine Learning in Whole-Body Magnetic Resonance Imaging for Detecting Metastases in Patients With Lung or Colon Cancer: A Diagnostic Test Accuracy Study.* Investigative Radiology, 2023: p. 10.1097.

3. Achterberg, H. *XNAT Python Client*. 9 June 2021]; Available from: https://xnat.readthedocs.io/en/latest/.

4. Fielding, R.T. and R.N. Taylor, *Principled design of the modern web architecture.* ACM Transactions on Internet Technology (TOIT), 2002. 2(2): p. 115-150.

5. Computational Imaging Lab, W.U., St Louis. *The XNAT API*. 2021 9 June 2021]; Available from: https://wiki.xnat.org/documentation/the-xnat-api.

6. Qaiser, T., et al. *Multiple Instance Learning with Auxiliary Task Weighting for Multiple Myeloma Classification*. in *International Conference on Medical Image Computing and Computer-Assisted Intervention*. 2021. Springer.

7. Health, N.I.o. *NIfTI*. 2013 [cited 2023 18 May 2023]; Available from: https://nifti.nimh.nih.gov/.

8. Li, X., et al., *The first step for neuroimaging data analysis: DICOM to NIfTI conversion.* Journal of neuroscience methods, 2016. 264: p. 47-56.

9. Doran, S.J., et al., *Integrating the OHIF Viewer into XNAT: Achievements, Challenges and Prospects for Quantitative Imaging Studies.* Tomography, 2022. 8(1).

10. Yushkevich, P.A., Y. Gao, and G. Gerig. *ITK-SNAP: An interactive tool for semi-automatic segmentation of multi-modality biomedical images*. in *2016 38th Annual International Conference of the IEEE Engineering in Medicine and Biology Society (EMBC)*. 2016. IEEE.

11. Gatys, L.A., A.S. Ecker, and M. Bethge. *Image style transfer using convolutional neural networks*. in *Proceedings of the IEEE conference on computer vision and pattern recognition*. 2016.

12. Creswell, A., et al., *Generative adversarial networks: An overview.* IEEE Signal Processing Magazine, 2018. 35(1): p. 53-65.

13. Karras, T., S. Laine, and T. Aila. *A style-based generator architecture for generative adversarial networks*. in *Proceedings of the IEEE/CVF Conference on Computer Vision and Pattern Recognition*. 2019.

14. Kalantar, R., et al., *Non-contrast CT synthesis using patch-based cycle-consistent generative adversarial network (Cycle-GAN) for radiomics and deep learning in the era of COVID-19.* Scientific Reports, 2023. 13(1): p. 10568.

15. Nyúl, L.G. and J.K. Udupa, *On standardizing the MR image intensity scale.* Magnetic Resonance in Medicine: An Official Journal of the International Society for Magnetic Resonance in Medicine, 1999. 42(6): p. 1072-1081.

16. Collaboration, I. *Interpolation*. 2022 2 January 2022]; Available from: https://ibsi.readthedocs.io/en/latest/02_Image_processing.html#interpolation.

17. Zwanenburg, A., et al., *The image biomarker standardization initiative: standardized quantitative radiomics for high-throughput image-based phenotyping.* Radiology, 2020. 295(2): p. 328-338.

Supplementary Figures

Supplementary Video 1. Complete 3-D data for reformatted and “composed” image series, illustrating a case where there are severe slice contiguity issues. Uncorrected, as here, the images would act as confounders for training ML algorithms.

Supplementary Video 2. Partially corrected 3-D data for the case shown in Supplementary Video 1. Where data are missing then the correction is imperfect.

Supplementary Figure 1. Detailed CONSORT diagram for MALIMAR study, augmented with data processing details and intermediate staging points. Phase 1 scans were for model training; Phase 2 were for human-in-the-loop testing of single time-point MRI scans for detection of active disease and Phase 3 scans were for human-in-the-loop testing of pre- and post-treatment MRI scan for detection of active disease as well as quantification of disease for detection of response.

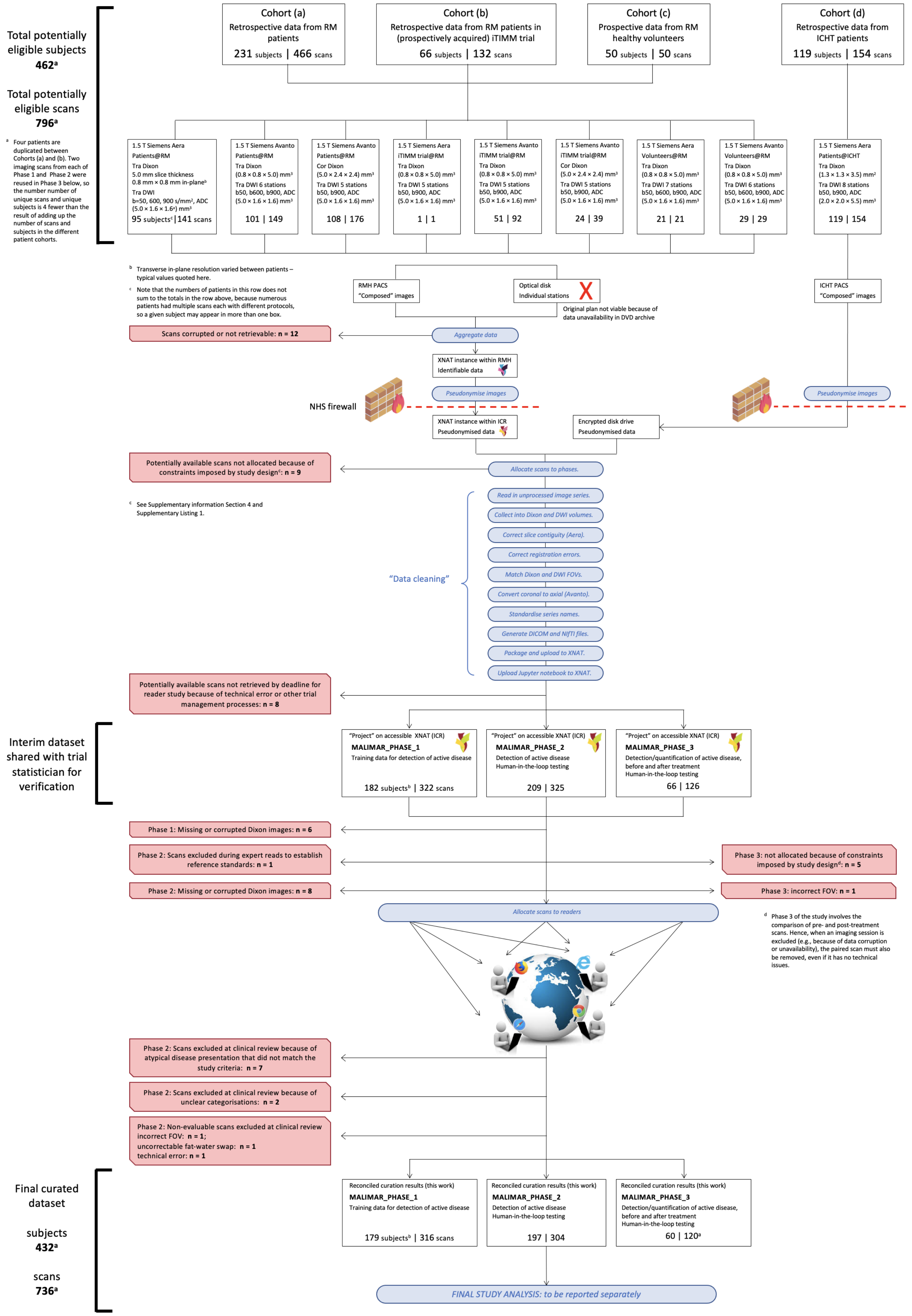

Supplementary Figure 2. “Poster frame” accompaniment to videos of complete 3-D data for reformatted “composed” image series, illustrating a case where there are severe slice contiguity issues. Uncorrected, the images would act as confounders for training ML algorithms. The original images can be partially corrected, but if data are missing then the correction is imperfect.

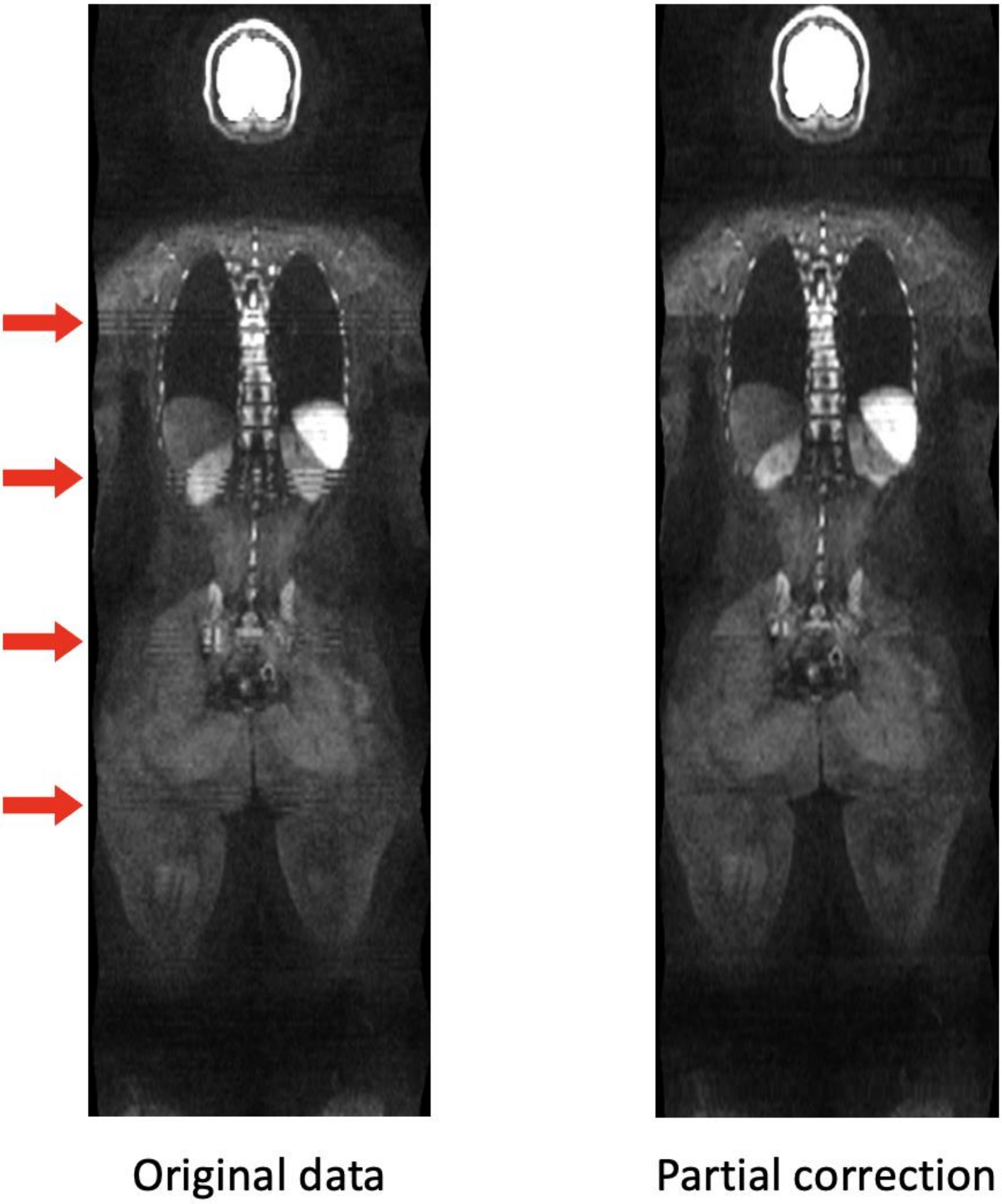

Supplementary Figure 3. Reformatted “composed” image series from a patient illustrating a case with completely misordered station data.

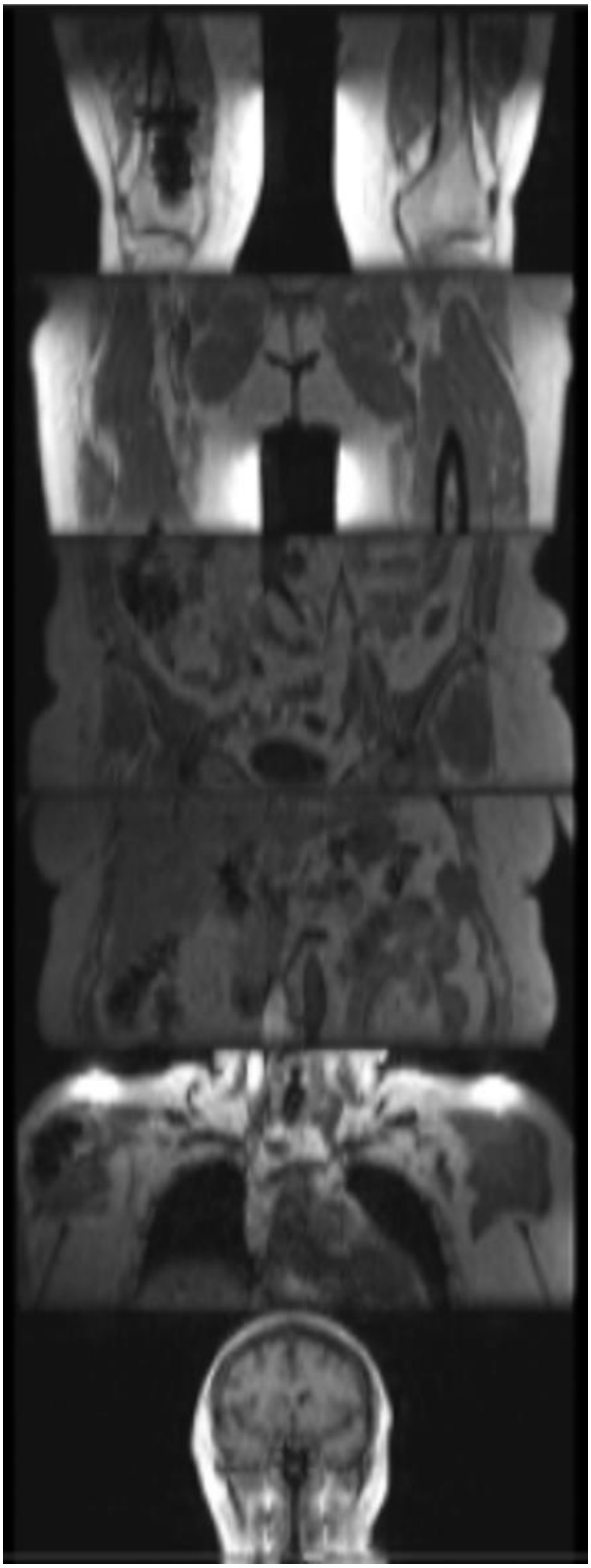

Supplementary Figure 4. Reformatted “composed” image series for two patients showing examples of the variations in field-of-view encountered.

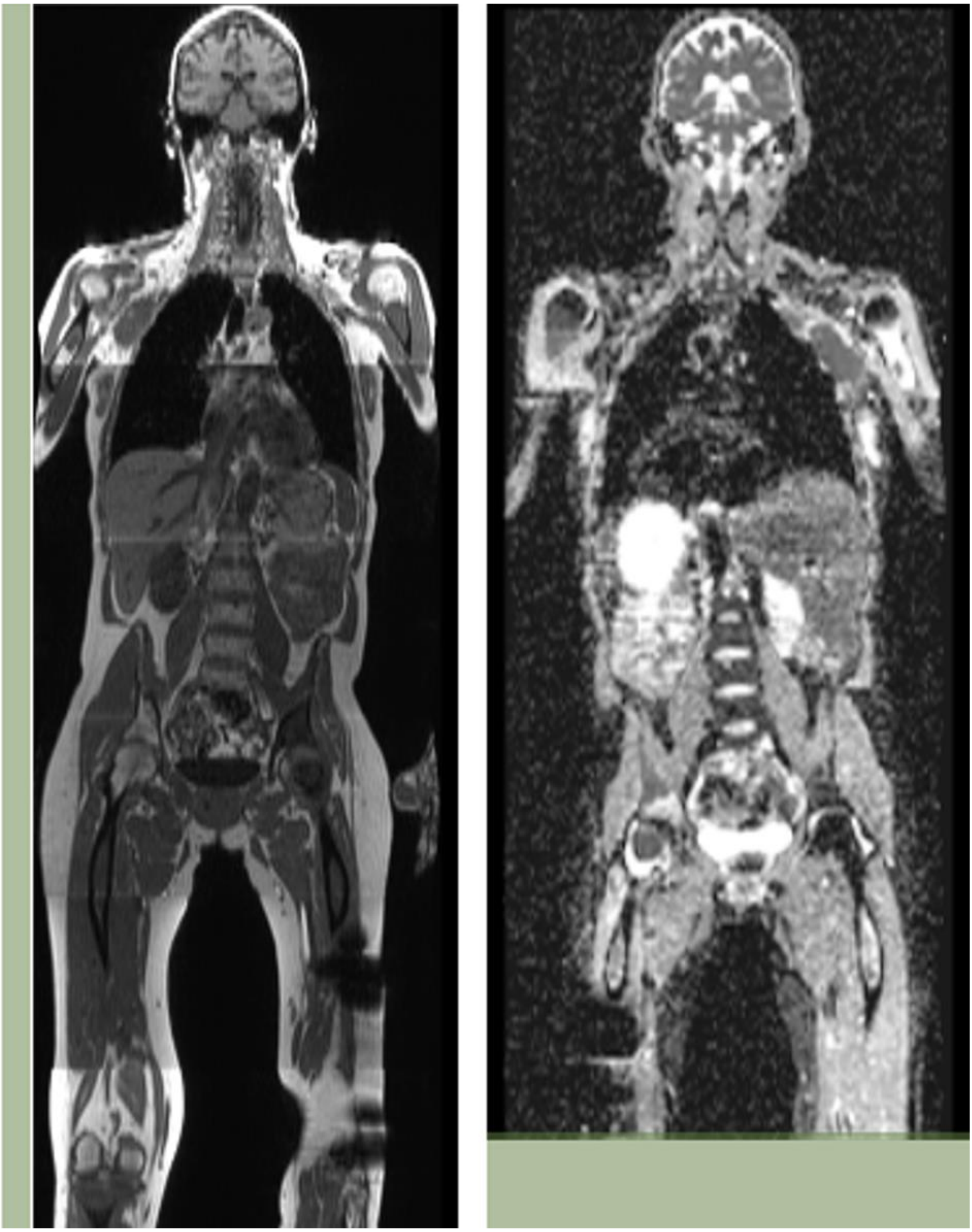

Supplementary Figure 5. Example of image with severe, but isolated, artefact due to presence of metal, with (inset) the corresponding b50 diffusion-weighted image. Other parts of the 3-D dataset may be suitable for machine learning. Note also (arrows) a local fat-water swap in the Dixon reconstruction.

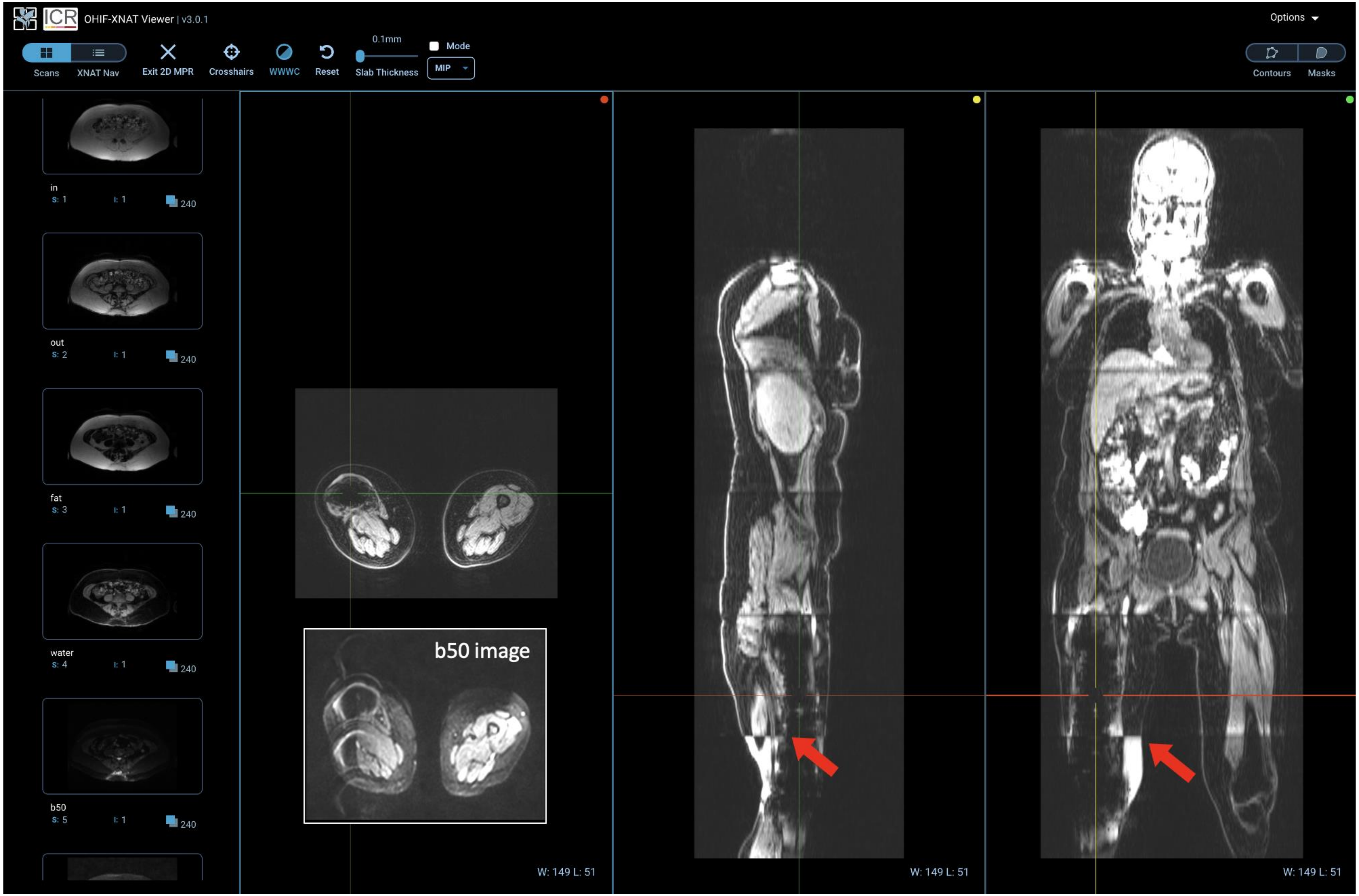

Supplementary Listing 1. Jupyter notebook illustrating the algorithm used for allocating imaging sessions to different trial phases and the way that this was made "self-documenting". Due to constraints of trial management, the RMH and ICHT data were assigned as separate processes.

MALIMAR Allocation ICH - Phase 1 & 2

Import Libraries to be used:

```
In [1]: import pandas as pd
        import numpy as np
        import random
        import seaborn as sns
        import warnings
        warnings.simplefilter(action='ignore')
```

Define a function that prints relevant and anonymised information when displaying tables:

```
In [2]: def print_df(df):
            return (df[['Anon Patient Name',
                        'DOS',
                        'Disease Category']].set_index(['Anon Patient Name','DOS'])).head(15)
```

Read in spreadsheet of all patient scans:

```
In [3]: pt_scans_all = pd.read_excel('patients_preallocation_ICH.xlsx')
        print('Number of cases:',len(pt_scans_all))

Number of cases: 154
```

First let's trim off those scan numbers from the trial IDs to form "Anonymised Patient Names"

Eg: ICH4820_001_1 --> ICH4820_001

```
In [4]: pt_scans_all['Anon Patient Name'] = pt_scans_all['Trial Number'].apply(lambda x: '_'.join(x.
```

```
In [5]: pt_scans_all.head()
Out[5]:
```

	DOS	AGE	Scan	Trial Number	Sex	Indication	Disease Pattern	Comments	Anon Patient Name
0	2017-05-18 00:00:00	72.9	1.0	ICH4820_001_1	M	Relapse	I	NaN	ICH4820_001
1	2017-05-18 10:46:00	72.5	1.0	ICH4820_002_1	F	Relapse	D	NaN	ICH4820_002
2	2017-05-18 17:29:00	48.6	1.0	ICH4820_003_1	F	Staging MM	D	NaN	ICH4820_003
3	2017-05-24 11:35:00	60.7	1.0	ICH4820_004_1	M	Relapse	F D	NaN	ICH4820_004
4	2019-10-23 00:00:00	63.0	2.0	ICH4820_004_2	M	Relapse	F D	NaN	ICH4820_004

Number of scans and patients:

```
In [6]: print('Number of Scans:',len(pt_scans_all))

Number of Scans: 154
```

```
In [7]: print('Number of Patients:',pt_scans_all['Anon Patient Name'].nunique())

Number of Patients: 119
```

MALIMAR Allocation - Phase 1 & 2

Disease categories:

Each scans is described by a 'Disease Pattern', the distribution of which are:

```
In [8]: pt_scans_all.groupby('Disease Pattern')['Anon Patient Name'].count()

Out[8]: Disease Pattern
        D             22
        D MN           1
        F             30
        F D           54
        F D PM        13
        F D PM EM      6
        F PM           7
        F PM EM        6
        I             15
        Name: Anon Patient Name, dtype: int64
```

Where F = Focal, D = Diffuse, I = Inactive, PM = Paramedullary, EM = Extramedullary, MN = Micronodullar and N = New Diagnosis (untreated).

While the above disease patterns are important variables that will be maintained throughout the course of the project, for the purpose of allocation these patterns will be assigned to D, F or I, using the following rules:

- Inactive simply remains as Inactive
- Any pattern containing Diffuse is assigned as Difuse
- Micromodullar is assigned as Diffuse
- All remaining patterns (F and F EM) assigned as Focal

Redefine categories into F, D or I:

```
In [9]: def assign_disease(pattern):
            if pattern == 'I':
                return 'I'
            elif 'D' in pattern:
                return 'D'
            elif 'MN' in pattern:
                return 'D'
            else:
                return 'F'
```

```
In [10]: pt_scans_all['Disease Category'] = pt_scans_all['Disease Pattern'].apply(lambda x: assign_di
```

With each scan now assigned a 'Disease Category' the totals are:

```
In [11]: pt_scans_all.groupby('Disease Category')['Anon Patient Name'].count()

Out[11]: Disease Category
         D    96
         F    43
         I    15
         Name: Anon Patient Name, dtype: int64
```

Allocate patients

Patients will be allocated to phases based on the disease pattern and date of their first WB-MRI scan involved in the MALIMAR study.

Order scans first by Anon Patient Name to group patients, then by Scan Date so each patient's scans are in chronological order:

```
In [12]: pt_scans = pt_scans_all.sort_values(by=['Anon Patient Name', 'DOS'])
         print_df(pt_scans)
Out[12]:
```

Anon Patient Name	DOS	Disease Category
ICH4820_001	2017-05-18 00:00:00	I
ICH4820_002	2017-05-18 10:46:00	D
ICH4820_003	2017-05-18 17:29:00	D
ICH4820_004	2017-05-24 11:35:00	D
	2019-10-23 00:00:00	D
ICH4820_005	2017-06-07 19:01:00	F
ICH4820_006	2017-06-14 18:44:00	F
	2019-05-21 00:00:00	D
ICH4820_007	2017-06-19 14:20:00	F
ICH4820_008	2017-06-21 15:53:00	D
ICH4820_009	2017-06-28 13:11:00	D
ICH4820_010	2017-06-28 15:43:00	F
	2019-06-23 00:00:00	F
ICH4820_011	2017-07-12 15:30:00	F
	2018-04-27 15:00:00	F

Keep first scan of each patient:

```
In [13]: pt_scans_first = pt_scans.drop_duplicates(subset='Anon Patient Name', keep="first")
         print_df(pt_scans_first)
Out[13]:
```

Anon Patient Name	DOS	Disease Category
ICH4820_001	2017-05-18 00:00:00	I
ICH4820_002	2017-05-18 10:46:00	D
ICH4820_003	2017-05-18 17:29:00	D
ICH4820_004	2017-05-24 11:35:00	D
ICH4820_005	2017-06-07 19:01:00	F
ICH4820_006	2017-06-14 18:44:00	F
ICH4820_007	2017-06-19 14:20:00	F
ICH4820_008	2017-06-21 15:53:00	D
ICH4820_009	2017-06-28 13:11:00	D
ICH4820_010	2017-06-28 15:43:00	F
ICH4820_011	2017-07-12 15:30:00	F
ICH4820_012	2017-07-13 16:18:00	D
ICH4820_013	2017-07-15 16:19:00	D
ICH4820_014	2017-07-19 15:56:00	F
ICH4820_015	2017-08-15 18:45:00	D

Number of patients in each category:

```
In [14]: pt_scans_first.groupby('Disease Category')['Anon Patient Name'].count()

Out[14]: Disease Category
         D    80
         F    29
         I    10
         Name: Anon Patient Name, dtype: int64
```

Create a new table (data frame) for each disease category:

```
In [15]: pt_scans_first_f = pt_scans_first[pt_scans_first['Disease Category'] == 'F']
         pt_scans_first_d = pt_scans_first[pt_scans_first['Disease Category'] == 'D']
         pt_scans_first_i = pt_scans_first[pt_scans_first['Disease Category'] == 'I']
```

For example the table for focal disease now looks like:

```
In [16]: print_df(pt_scans_first_f)
Out[16]:
```

Anon Patient Name	DOS	Disease Category
ICH4820_005	2017-06-07 19:01:00	F
ICH4820_006	2017-06-14 18:44:00	F
ICH4820_007	2017-06-19 14:20:00	F
ICH4820_010	2017-06-28 15:43:00	F
ICH4820_011	2017-07-12 15:30:00	F
ICH4820_014	2017-07-19 15:56:00	F
ICH4820_016	2017-08-23 16:29:00	F
ICH4820_019	2017-09-13 17:56:00	F
ICH4820_034	2018-02-05 16:21:00	F
ICH4820_036	2018-02-19 13:03:00	F
ICH4820_041	2018-03-21 15:39:00	F
ICH4820_045	2018-04-16 10:34:00	F
ICH4820_057	2018-06-21 14:18:00	F
ICH4820_059	2018-08-14 13:23:00	F
ICH4820_064	2018-09-27 17:31:00	F

Sort first scans in each disease category by date:

```
In [17]: pt_scans_first_f = pt_scans_first_f.sort_values('DOS')
         pt_scans_first_d = pt_scans_first_d.sort_values('DOS')
         pt_scans_first_i = pt_scans_first_i.sort_values('DOS')
```

For example the table for focal disease now looks like:

```
In [18]: print_df(pt_scans_first_f)
Out[18]:
```

Anon Patient Name	DOS	Disease Category
ICH4820_005	2017-06-07 19:01:00	F
ICH4820_006	2017-06-14 18:44:00	F
ICH4820_007	2017-06-19 14:20:00	F
ICH4820_010	2017-06-28 15:43:00	F
ICH4820_011	2017-07-12 15:30:00	F
ICH4820_014	2017-07-19 15:56:00	F
ICH4820_016	2017-08-23 16:29:00	F
ICH4820_019	2017-09-13 17:56:00	F
ICH4820_034	2018-02-05 16:21:00	F
ICH4820_036	2018-02-19 13:03:00	F
ICH4820_041	2018-03-21 15:39:00	F
ICH4820_045	2018-04-16 10:34:00	F
ICH4820_057	2018-06-21 14:18:00	F
ICH4820_059	2018-08-14 13:23:00	F
ICH4820_064	2018-09-27 17:31:00	F

Allocate patients (by first scan) to phase 1 and 2:

Within each of the disease categories (F, D and I) the patients are allocated to phases 1 and 2 on an alternating basis. All even rows (0, 2, 4 ..) are allocated to phase 1 and all odd rows (1, 3, 5 ..) are allocated to phase 2:

```
In [19]: pt_scans_first_f_p1 = pt_scans_first_f.iloc[(range(0, len(pt_scans_first_f), 2))]
         pt_scans_first_d_p1 = pt_scans_first_d.iloc[(range(0, len(pt_scans_first_d), 2))]
         pt_scans_first_i_p1 = pt_scans_first_i.iloc[(range(0, len(pt_scans_first_i), 2))]

         pt_scans_first_f_p2 = pt_scans_first_f.iloc[(range(1, len(pt_scans_first_f), 2))]
         pt_scans_first_d_p2 = pt_scans_first_d.iloc[(range(1, len(pt_scans_first_d), 2))]
         pt_scans_first_i_p2 = pt_scans_first_i.iloc[(range(1, len(pt_scans_first_i), 2))]
```

For example the patients allocated to phase 1 based on their first scan presenting focal disease:

```
In [20]: print_df(pt_scans_first_f_p1)
Out[20]:
```

Anon Patient Name	DOS	Disease Category
ICH4820_005	2017-06-07 19:01:00	F
ICH4820_007	2017-06-19 14:20:00	F
ICH4820_011	2017-07-12 15:30:00	F
ICH4820_016	2017-08-23 16:29:00	F
ICH4820_034	2018-02-05 16:21:00	F
ICH4820_041	2018-03-21 15:39:00	F
ICH4820_057	2018-06-21 14:18:00	F
ICH4820_064	2018-09-27 17:31:00	F
ICH4820_076	2019-01-17 00:00:00	F
ICH4820_094	2019-06-09 00:00:00	F

Number of patients in each phase grouped by the disease category of their first scan:

```
In [21]: print('Diffuse Patients:','Phase 1 =',len(pt_scans_first_d_p1),', Phase 2 = ',len(pt_scans_f

Diffuse Patients: Phase 1 = 40 , Phase 2 =  40
```

```
In [22]: print('Focal Patients:','Phase 1 =',len(pt_scans_first_f_p1),', Phase 2 = ',len(pt_scans_fir

Focal Patients: Phase 1 = 15 , Phase 2 =  14
```

```
In [23]: print('Inactive Patients:','Phase 1 =',len(pt_scans_first_i_p1),', Phase 2 = ',len(pt_scans_

Inactive Patients: Phase 1 = 5 , Phase 2 =  5
```

Allocate scans to phase 1 and 2

The patients have now been allocated to phases 1 and 2, and the hospital numbers are used to allocate all scans:

Extract anon patient names for each phase and disease category:

```
In [24]: pn_f_p1 = set(pt_scans_first_f_p1['Anon Patient Name'])
         pn_d_p1 = set(pt_scans_first_d_p1['Anon Patient Name'])
         pn_i_p1 = set(pt_scans_first_i_p1['Anon Patient Name'])

         pn_f_p2 = set(pt_scans_first_f_p2['Anon Patient Name'])
         pn_d_p2 = set(pt_scans_first_d_p2['Anon Patient Name'])
         pn_i_p2 = set(pt_scans_first_i_p2['Anon Patient Name'])
```

Allocate all scans assosciated with the patients in phase 1 and 2:

```
In [25]: pt_scans_p1 = pt_scans[pt_scans['Anon Patient Name'].isin(pn_f_p1 | pn_d_p1 | pn_i_p1)]
         pt_scans_p2 = pt_scans[pt_scans['Anon Patient Name'].isin(pn_f_p2 | pn_d_p2 | pn_i_p2)]
```

Number of scans in each phase:

```
In [26]: print('Phase 1 scans = ', len(pt_scans_p1), ', Phase 2 scans = ', len(pt_scans_p2))

Phase 1 scans =  80 , Phase 2 scans =  74
```

Distribution Across Phases

Disease Pattern and Category

```
In [27]: pt_scans_p1.groupby('Disease Pattern')['Anon Patient Name'].count()

Out[27]: Disease Pattern
         D              9
         D MN           1
         F             18
         F D           26
         F D PM         7
         F D PM EM      5
         F PM           5
         F PM EM        2
         I              7
         Name: Anon Patient Name, dtype: int64
```

```
In [28]: pt_scans_p1.groupby('Disease Category')['Anon Patient Name'].count()

Out[28]: Disease Category
         D    48
         F    25
         I     7
         Name: Anon Patient Name, dtype: int64
```

Number of scans in each disease pattern and category for phase 2:

```
In [29]: pt_scans_p2.groupby('Disease Pattern')['Anon Patient Name'].count()

Out[29]: Disease Pattern
         D             13
         F             12
         F D           28
         F D PM         6
         F D PM EM      1
         F PM           2
         F PM EM        4
         I              8
         Name: Anon Patient Name, dtype: int64
```

```
In [30]: pt_scans_p2.groupby('Disease Category')['Anon Patient Name'].count()

Out[30]: Disease Category
         D    48
         F    18
         I     8
         Name: Anon Patient Name, dtype: int64
```

Output Spreadsheets:

Create spreadsheet for each phase:

```
In [31]: pt_scans_p1.to_excel('patients_phase1_ICH.xlsx',index = False)
         pt_scans_p2.to_excel('patients_phase2_ICH.xlsx',index = False)
```

Create spreadsheet with both phases with phase in column:

```
In [32]: pt_scans_p1['Phase'] = 1
         pt_scans_p2['Phase'] = 2
         pt_scans = pd.concat([pt_scans_p1,pt_scans_p2],sort = False)
         pt_scans.to_excel('patients_allocated.xlsx',index = False)
```

```
In [33]: !jupyter nbconvert --to html MALIMAR_Allocation_ICH.ipynb

[NbConvertApp] Converting notebook MALIMAR_Allocation_ICH.ipynb to html
[NbConvertApp] Writing 402739 bytes to MALIMAR_Allocation_ICH.html
```

Supplementary Listing 2. Jupyter notebook illustrating the algorithm used for cleaning imaging sessions and the way that this was made “self-documenting”. Note that the script refers to a set of underlying Python objects that were developed using a traditional Python coding methodology with the PyCharm IDE. At the point marked *, the Jupyter script launches an interactive editing tool to correct for image shifts. Arrows represent the order of the process flow.

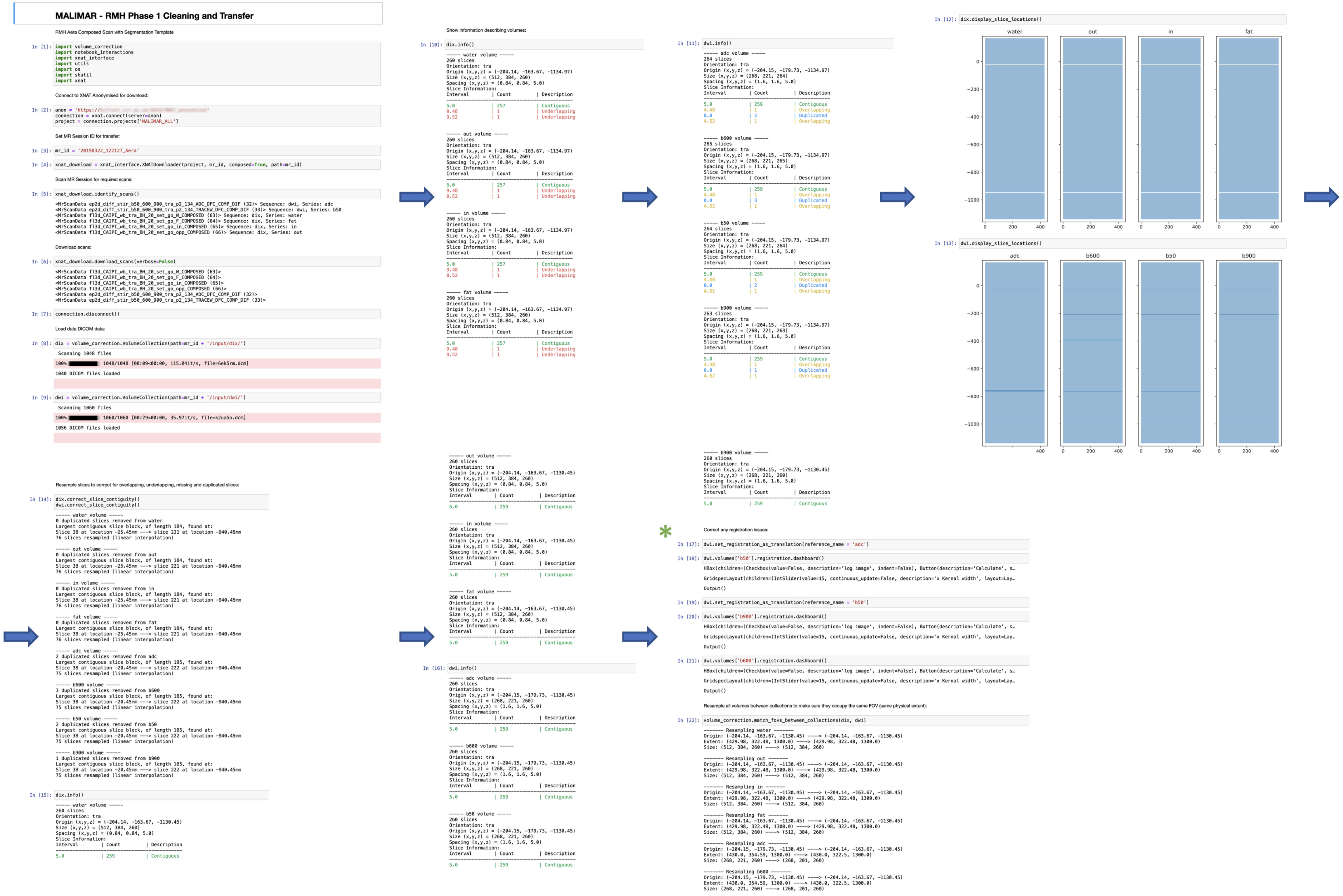

```
------- Resampling b50 -------
Origin: (-204.15, -179.73, -1130.45) ----> (-204.14, -163.67, -1130.45)
Extent: (430.0, 354.59, 1300.0) ----> (430.0, 322.5, 1300.0)
Size: (268, 221, 260) ----> (268, 201, 260)

------- Resampling b900 -------
Origin: (-204.15, -179.73, -1130.45) ----> (-204.14, -163.67, -1130.45)
Extent: (430.0, 354.59, 1300.0) ----> (430.0, 322.5, 1300.0)
Size: (268, 221, 260) ----> (268, 201, 260)
```

Export volumes as NIfTI and DICOM, modifying DICOM tags as necesary:

```
In [23]: dix.write_to_nifti()
         dix.write_to_dicom()
         dwi.write_to_nifti()
         dwi.write_to_dicom()

water saved as NIfTI file
out saved as NIfTI file
in saved as NIfTI file
fat saved as NIfTI file
"water" image volume saved as DICOM series
"out" image volume saved as DICOM series
"in" image volume saved as DICOM series
"fat" image volume saved as DICOM series
adc saved as NIfTI file
b600 saved as NIfTI file
b50 saved as NIfTI file
b900 saved as NIfTI file
"adc" image volume saved as DICOM series
"b600" image volume saved as DICOM series
"b50" image volume saved as DICOM series
"b900" image volume saved as DICOM series
```

Connect to XNAT and download existing segmentations:

```
In [24]: colab = 'https://[illegible]'
         connection = xnat.connect(server=colab)
         project = connection.projects['MALIMAR_PHASE1']

In [25]: xnat_upload = xnat_interface.XNATUploader(project, path=mr_id, mr_session_id=mr_id)

In [26]: seg_paths = xnat_interface.download_segmentations(mr_session=xnat_upload.mr_session, path=mr_id)

Downloading dixon_seg_20200318_173142.nii.gz segmentation
Downloading diffusion_seg_20200318_173207.nii.gz segmentation

In [27]: seg_paths

Out[27]: {'dixon': '20190322_122127_Aera/dixon_seg.nii.gz',
          'diffusion': '20190322_122127_Aera/diffusion_seg.nii.gz'}
```

Check volumes and segmentation alignmnent in ITK-SNAP:

```
In [28]: dix.open_in_itk_snap(seg_path=seg_paths['dixon'])

In [29]: dwi.open_in_itk_snap(seg_path=seg_paths['diffusion'])
```

Zip DICOMs for upload:

```
In [30]: shutil.make_archive(mr_id + '/output/dicom', 'zip', mr_id + '/output/dicom')

Out[30]: '/Users/tbarfoot/Documents/MALIMAR_data_cleaning/20190322_122127_Aera/output/dicom.zip'
```

Check volumes and segmentation alignmnent in ITK-SNAP:

```
In [28]: dix.open_in_itk_snap(seg_path=seg_paths['dixon'])

In [29]: dwi.open_in_itk_snap(seg_path=seg_paths['diffusion'])
```

Zip DICOMs for upload:

```
In [30]: shutil.make_archive(mr_id + '/output/dicom', 'zip', mr_id + '/output/dicom')

Out[30]: '/Users/tbarfoot/Documents/MALIMAR_data_cleaning/20190322_122127_Aera/output/dicom.zip'
```


Replace DICOM and NIfTI files:

```
In [31]: xnat_upload.replace_dicom()

Deleting Scans (DICOM and NIfTI) from https://[illegible]
<MrScanData in (1)> deleted
<MrScanData out (2)> deleted
<MrScanData fat (3)> deleted
<MrScanData water (4)> deleted
<MrScanData b50 (5)> deleted
<MrScanData b600 (6)> deleted
<MrScanData b900 (7)> deleted
<MrScanData adc (8)> deleted
Uploading DICOMs to https://[illegible]

In [32]: xnat_upload.upload_nifti()

-- Uploading NIFTIs --
Uploading in.nii.gz
Uploading out.nii.gz
Uploading fat.nii.gz
Uploading water.nii.gz
Uploading b50.nii.gz
Uploading b600.nii.gz
Uploading b900.nii.gz
Uploading adc.nii.gz
```

Add sex of patient to XNAT:

```
In [33]: sex = dix.volumes['in'].dcm_header.PatientSex

In [34]: xnat_upload.mr_session.subject.demographics.gender = sex
```

Save and upload this notebook:

```
In [35]: !jupyter nbconvert --output-dir={mr_id} --to html --output cleaning_notebook cleaning_notebook-rmh_aera_c_seg.ipynb

[NbConvertApp] Converting notebook cleaning_notebook-rmh_aera_c_seg.ipynb to html
[NbConvertApp] Writing 40872468 bytes to 20190322_122127_Aera/cleaning_notebook.html

In [36]: xnat_upload.upload_cleaning_notebook()
```

Close connection and remove temp files:

```
In [37]: connection.disconnect()

In [38]: shutil.rmtree(mr_id, ignore_errors=True)

In [39]: _ = os.system('say "Upload complete!"')
```
